# Dedifferentiated Chondrosarcoma from Molecular Pathology to Current Treatment and Clinical Trials

**DOI:** 10.3390/cancers15153924

**Published:** 2023-08-01

**Authors:** Weronika Zając, Julia Dróżdż, Weronika Kisielewska, Weronika Karwowska, Monika Dudzisz-Śledź, Agnieszka E. Zając, Aneta Borkowska, Anna Szumera-Ciećkiewicz, Bartłomiej Szostakowski, Piotr Rutkowski, Anna M. Czarnecka

**Affiliations:** 1 Department of Soft Tissue/Bone Sarcoma and Melanoma, Maria Sklodowska Curie National Research Institute of Oncology, 02-781 Warsaw, Polandmonika.dudzisz-sledz@pib-nio.pl (M.D.-Ś.); agnieszka.zajac@pib-nio.pl (A.E.Z.); aneta.borkowska@pib-nio.pl (A.B.); bartlomiej.szostakowski@pib-nio.pl (B.S.); piotr.rutkowski@pib-nio.pl (P.R.); 2 Faculty of Medicine, Medical University of Warsaw, 02-091 Warsaw, Poland; 3 Department of Pathology, Maria Sklodowska Curie National Research Institute of Oncology, 02-781 Warsaw, Poland; anna.szumera-cieckiewicz@pib-nio.pl

**Keywords:** chondrosarcoma dedifferentiated, targeted treatment, immunohistochemistry, palliative treatment, pathomorphology

## Abstract

**Simple Summary:**

Dedifferentiated chondrosarcoma is a rare type of cancer that is very aggressive and has a poor prognosis with poor survival rates. This disease can affect anyone of any age, but it is usually diagnosed among people 50 years of age or older. There is no standard treatment available; usually it is based on surgery, however most patients are diagnosed with an advanced stage when radical treatment is not possible. We present the most up–to–date data on genetics, diagnostic procedures, and treatment options for localised and advanced diseases.

**Abstract:**

Dedifferentiated chondrosarcoma (DDCS) is a rare subtype of chondrosarcoma, a primary cartilaginous malignant neoplasm. It accounts for up to 1–2% of all chondrosarcomas and is generally associated with one of the poorest prognoses among all chondrosarcomas with the highest risk of metastasis. The 5-year survival rates range from 7% to 24%. DDCS may develop at any age, but the average presentation age is over 50. The most common locations are the femur, pelvis humerus, scapula, rib, and tibia. The standard treatment for localised disease is surgical resection. Most patients are diagnosed in unresectable and advanced stages, and chemotherapy for localised and metastatic dedifferentiated DDCS follows protocols used for osteosarcoma.

## 1. Introduction

Dedifferentiated chondrosarcoma (DDCS) is a rare subtype of chondrosarcoma that is a primary cartilaginous malignant neoplasm [1]. It is usually characterised by two distinctive histopathological components with a clear demarcation line [2]. The first part is a low-grade tumour which, due to the deposition of non-osseous hyaline cartilage matrix, is considered to be chondrosarcoma, closely intersected with the second component, high-grade non-cartilaginous sarcoma tumour [3]. This is a definition proposed by Dahlin and Beabout in 1971 and is still relevant today [4]. 

DDCS can arise spontaneously, but even in half of the cases, the high-grade component emerges from a pre-existing low-grade chondrosarcoma [2]. It means that the second part results from the dedifferentiation of some of the chondrosarcoma cells that lost a phenotype of cartilaginous cells and cells that can produce cartilage [5]. Dedifferentiated chondrosarcomas primarily develop as the central subtype, with only a small percentage classified as peripheral dedifferentiated chondrosarcomas. These peripheral cases typically arise from low-grade chondrosarcomas that originate from the cartilage cap of a preexisting benign osteochondroma. Osteochondroma refers to a benign bony outgrowth covered by a cartilaginous cap, with the solitary sporadic form being approximately six times more common than the occurrence within the context of multiple osteochondromas. In both solitary and multiple osteochondromas, the genomic level is affected by mutations and/or deletions in the EXT1 and/or EXT2 genes [6]. The high-grade component can show features of various sarcomas. The most frequent manifestation is osteosarcoma (OSC), but also often, it is a fibrosarcoma (FS), malignant fibrous histiocytoma (MFH), or it remains undifferentiated, pleomorphic tissue. There have been reports of leiomyosarcoma (LMS), clear-cell chondrosarcoma (CCCS), mesenchymal chondrosarcoma (MCS) or rhabdomyosarcoma (RMS), but those manifestations are rare [7]. DDCS is generally associated with one of the poorest prognoses among all types of chondrosarcomas. Furthermore, it has one of the highest chances of metastasis and local recurrence even after treatment with a wide-margin surgery and adjuvant systemic therapy [8,9]. 

Due to the rarity of this disease and its aggressive nature, it is vital to continue research on DDCS to improve diagnostics and find more efficient therapies for adjuvant setting and metastatic disease. A compendium of information on the disease is also essential because it would enable rapid diagnosis and facilitate efficient decision-making. In this work, we summarise the current state of knowledge of DDCS. We also present the relevant data related to these tumours’ epidemiology, diagnostic criteria, manifestations, pathology, and genetics. Moreover, we discuss the available treatment methods with the potential candidates for future therapies and ongoing clinical trials.

## 2. Epidemiology

Although chondrosarcoma makes up approximately 20% of primary bone malignancies and is currently the second most common bone sarcoma, DDCS occurs more rarely, accounting for only 1–2% of all bone malignancies, and 10 to 20% of all chondrosarcomas [10]. It can arise from existing chondrosarcoma, and the risk of differentiation of any conventional chondrosarcoma (CCS) case is estimated to be 7–20% [11,12]. Dedifferentiation mostly occurs in conventional central chondrosarcoma, constituting 10–15% of all cases that result in manifestation of the malignancy. It can also occur in peripheral chondrosarcoma; however, the chance of this event is extremely low. It is due to a rare incidence of peripheral chondrosarcoma [13,14]. Although DDCS can arise at any age, the average patient is 50 or older. The median age is described as around 60 years. There is a slightly greater incidence of this malignancy among elderly men than women [7,8]. The male-to-female ratio is 1.5 to 1 in DDCS [15]. DDCS is associated with a poor prognosis and an aggressive course, leading to a 5-year survival rate of 7% to only a maximum of 24%, with median survival ranging from 7 to 15 months [9,16]. Localisation in the axial skeleton, especially in the pelvic bone, is associated with a poorer prognosis, probably due to the greater difficulty of operating in that area and not achieving radical surgical resection. Similarly, the larger size of the tumours (more than 8 cm) leads to a lower 5-year survival rate due to the difficulty in achieving the radical margin [17]. Almost half of the patients suffer from local recurrence (LR) in that location [18]. The development is another poor prognostic factor, with a 5-year survival rate equal to a range of as little as 5 months to 15 months [19,20]. Other factors related to poor prognosis are patients aged 60 years or above and pathological fractures [10,17]. According to research, if the non-cartilaginous component is a fibrosarcoma, it is related to a greater risk of metastasis and therefore, even lower survival rate [21].

### Dedifferentiated Chondrosarcoma in Ollier Disease and Mafucci Syndrome

Ollier disease (OD) and Mafucci syndrome (MS) are characterised by the appearance of multiple (more than 3) that are benign expansions of cartilage. They are both rare and not inherited disorders. In OD, the neoplasm appears mainly in the appendicular skeleton of one side of the body, whereas in MS, the tumours are bilateral with accidental distribution. Furthermore, in MS, enchondroma is often followed by vascular malignancies [22]. The chance of developing secondary chondrosarcoma from primary enchondroma is approximately equal to 25% in patients with OD, while more than 50% of patients with MF have secondary malignancy [23]. Various research suggests a greater probability of dedifferentiation of the tumour as 6% of patients will develop aggressive grade 3 secondary chondrosarcoma [11]. Aycan et al. reported a secondary chondrosarcoma with lung metastasis that radiologically and histologically showed features of DDCS. The incidence of metastasis in such cases is almost always equal to 100%. Additionally, on average, the manifestation of DDCS can appear 10 years earlier [24]. 

## 3. Diagnostics

### 3.1. Location and Metastasis

DDCS is a disease of bone tissue therefore, it occurs in the skeletal system, primarily in the appendicular skeleton, with a greater chance of it appearing in the lower limb [25]. The most popular locations are femur (over 35% of cases), pelvis (up to 29% of cases), humerus (16%), as well as scapula (6%), rib (6%), and tibia (5%). In particular, femur is the most common location identified, in some case series constituting up to 44.5% of patients, while the humerus is the third most common location, but also in other types of bones, most primarily pelvic bones (second most common location identified in 22.2% of patients) and scapulae [9,16,26]. More frequently, it is located in the proximal rather than distal part of the previously mentioned long bones [27]. There has been a record of cases in which DDCS was localised in the ribs and phalanges of the hand and foot, but those locations are extremely rare [26]. Most of the neoplastic change is found in the medullary cavity, but approximately one in five cases are situated externally on the surface of the bone [4,28]. Studies show that approximately 8 to 40% of patients with DDCS develop distant metastatic sites, and metastasis is often present during diagnosis. The malignancy frequently metastasises to the lungs (15%), other sites (8%), and skeletal system (2%) [25,29]. Advanced age, higher grade of malignancies, larger tumour size (>8 cm), and localisation in the pelvis are established risk factors for distant metastasis [25,30]. Metastatic tissue is usually dedifferentiated and resembles the same type of tissue located at the primary site of the tumour [29].

### 3.2. Size

In DDCS, the mass of the tumour is usually much greater than in conventional chondrosarcoma due to the second, non-cartilaginous component. The size of the tumour mass varies from 5 mm to 36 mm [18] or even to 460 mm [31]. The size of its constitutive components is also diverse and difficult to predict, especially in terms of the high-grade component, which is increasing with the progress of the disease [32]. On radiological images, the average size of the greatest dimension of newly diagnosed chondrosarcoma is reported to be 9.5 cm [27]. The dedifferentiated component can be either may be small or account for half of the tumour mass; however, no relationship between total size and percentage of differentiation was observed [33].

### 3.3. Symptoms

Symptoms are usually nonspecific and present themselves later as the disease progresses. At first, there may be no external manifestation of the malignancy. Usually during the progression of the disease, such symptoms as pain, swelling, edema, and paraesthesia are described in the tumour’s proximity. The pain is constantly increasing over time [34]. A palpable mass can be felt only in one third of patients, making it more difficult to recognise the tumour. The bone that weakens with the development of the disease is prone to frequent pathological fractures and occurs in half of patients with DDCS. The tumour itself is often detected when a patient is hospitalised for such a problem [16,25]. Fracture leads to the development of hematoma and spread of tumour cells but does not increase the risk of metastasis, it only indicates the higher grade and/or size of the tumour [33]. 

### 3.4. Methods of Diagnosis

The two most common diagnostic methods include radiological and histological procedures. Among the former, mostly radiography, contrast-enhanced computed tomography (CT) and magnetic resonance imagining (MRI) are used. There has been no evidence of using position emission tomography (PET) to diagnose malignancy. The most common method of histological diagnosis is by biopsy, after which macroscopic and microscopic examination is performed. It is used to confirm the diagnosis, identify subtypes, and advance the disease because radiological images of conventional chondrosarcoma and its dedifferentiated subtype are difficult to differentiate in 50% of cases [20].

#### 3.4.1. Radiological Criterium

Most images show a mass divided into two parts corresponding to previously described phases of tumour development [32]. In most cases, the radiographic characteristics of dedifferentiated chondrosarcoma typically resemble intermediate to high-grade chondrosarcoma. However, in a few cases, they exhibit similar features to low-grade chondrosarcoma. Additionally, approximately 10–15% of cases do not exhibit any radiographic signs indicating the presence of a chondroid matrix or an underlying chondral tumour. Instead, some of these cases exhibit features indicative of osteosarcoma [35,36,37].

**Figure 1:**
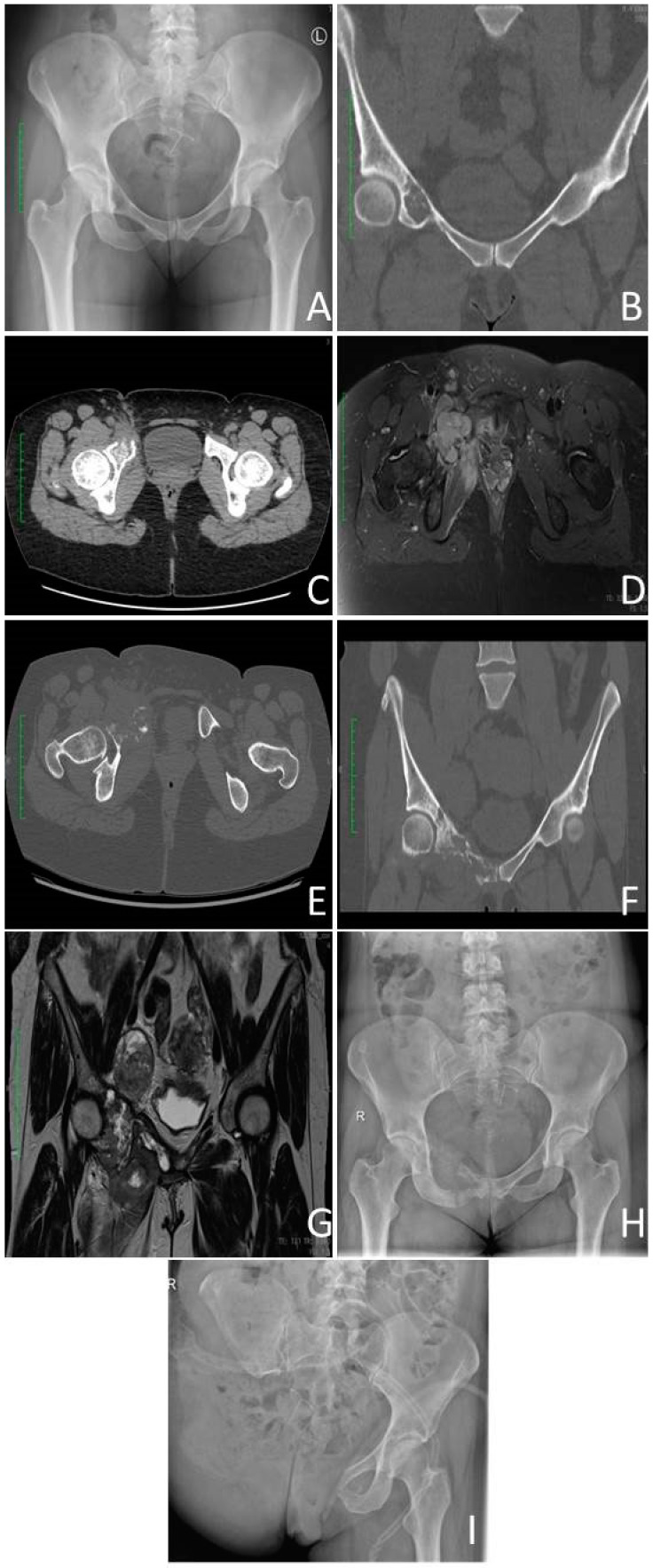
Dedifferentiated high-grade chondrosarcoma of right pubic bone. Initial presentation of the chondral lesion located in the right pubic bone, abutting acetabulum (October 2021): (**A**)-Anterior–posterior (AP) X-Ray view of the pelvis; (**B**) Coronal computed tomography (CT) view. (**C**)-CT Axial view—growing lesion on the follow up CT did not alert necessary attention (January 2022). (**D**) Contrast-enhanced magnetic resonance imaging (MRI) with fat saturation showing enlarging lesion on the follow up (August 2022). (**E**,**F**) Coronal and axial images of the progressing right pubic lesion on contrast enhanced CT scan. (**G**,**H**) Preoperative contrast-enhanced MRI coronal view scan and X-ray AP view showing lesion of the right pubic bone that progressed over 14 months (November 2022). (**I**). Postoperative image after right hind-quarter amputation.

**Figure 2:**
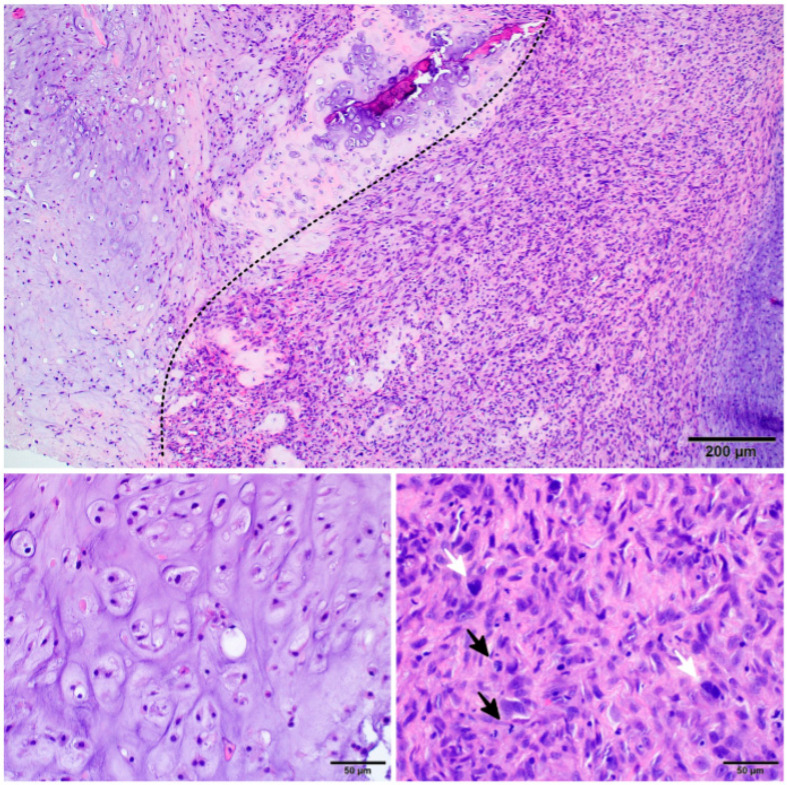
Histopathological image of dedifferentiated chondrosarcoma: the transition between components of low and high grade is clear-cut (the dotted line); the low-grade chondrosarcoma is cartilaginous and usually appears as grade 1 or 2; the high-grade dedifferentiated part presents as high-grade undifferentiated sarcoma with high mitotic activity (black arrow) and high pleomorphism (white arrow).

**Figure 3:**
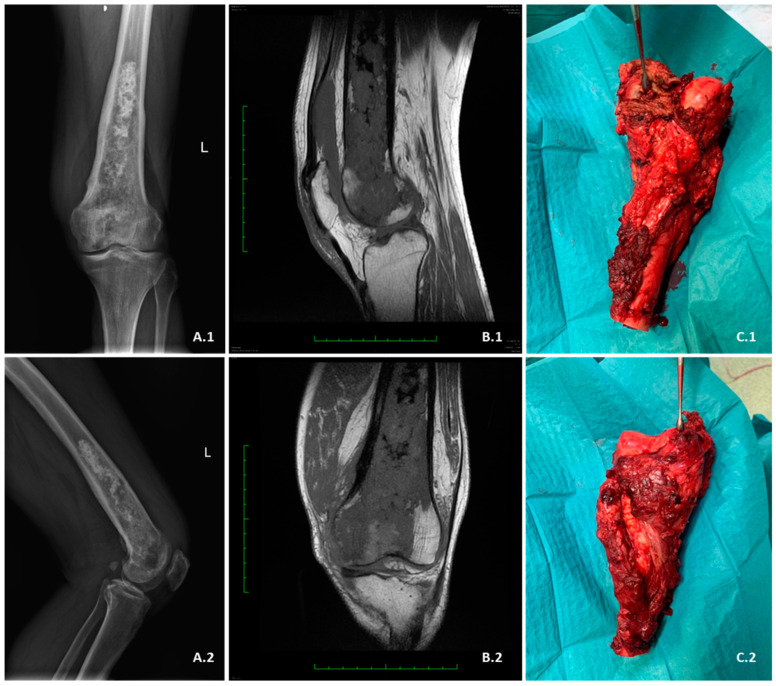
WHO G3 dedifferentiated chondrosarcoma of distal femur. (**A.1**,**A.2**)–AP/lateral (LAT) X-ray preoperative views. (**B.1**) Saggital fat saturated T1 MRI view. (**B.2**) Coronal fat saturated T1 MRI view. (**C.1**,**C.2**) Intraoperative views of the resected distal femur.

**Figure 4:**
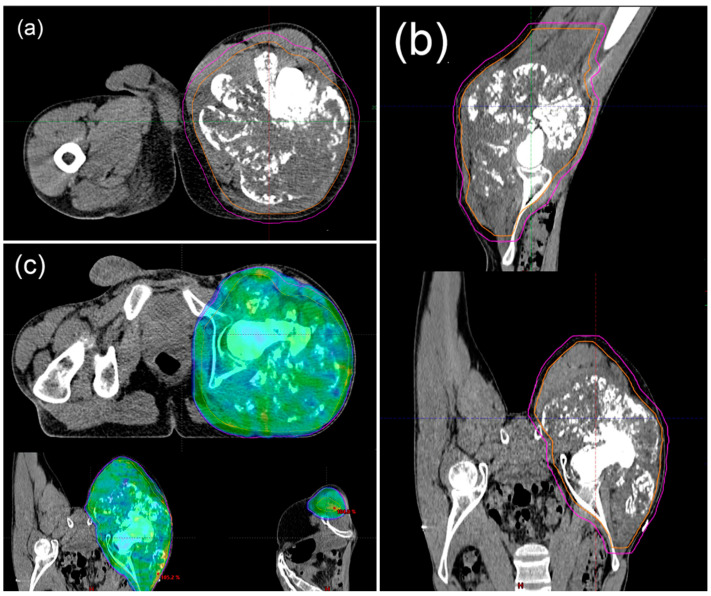
Nineteen-year-old patient diagnosed with dedifferentiated chondrosarcoma on the right thigh with lung metastases. Status after seven courses of chemotherapy with significant regression of lung lesions. Patient received radiotherapy for a lesion on the right thigh. Radiation therapy CT planning (**a**,**b**). Radiation therapy plan (**c**).

**Figure 5:**
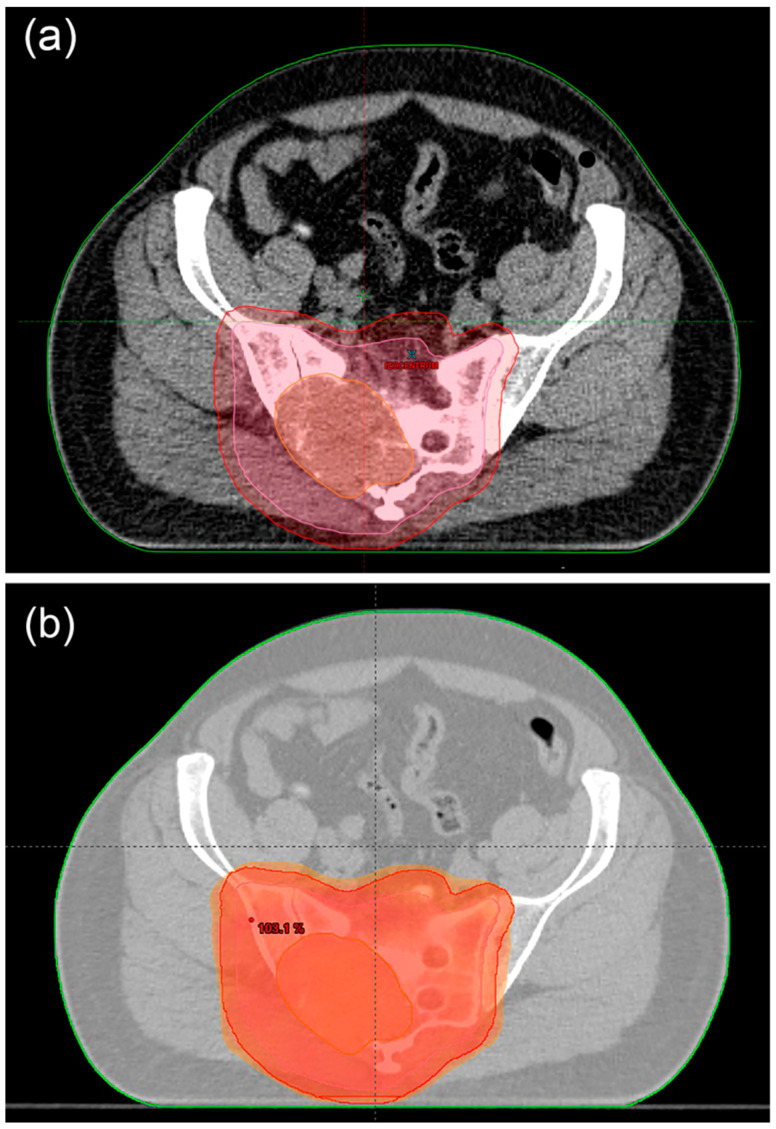
Fifty-year-old patient diagnosed with dedifferentiated chondrosarcoma of the left scapula, at the stage of spread to the sacrum. Received radiation therapy for sacral metastasis. Radiation therapy CT planning (**a**). Radiation therapy plan (**b**).

The radiographic characteristics of dedifferentiated chondrosarcomas consist of a tumour bimorphism, which involves aggressive destruction of the bone accompanied by extension into the surrounding soft tissues (Figure 1, Figure 2, Figure 3, Figure 4 and Figure 5). These features are typically associated with an underlying cartilaginous lesion [37]. The lesion that produces hyaline cartilage is presented as a calcified area with extraosseous mineralisation of its matrix. Next, there is a lytic area of gray-to-soft-white tissue representing non-cartilaginous malignancy. The transition between the two parts should be clear and abrupt highlighting the visible demarcation line between both parts [2]. On radiographs and CT scans, a dual characteristic appearance has been observed in approximately 30% and 50% of cases, respectively. This appearance includes the presence of an unmineralised tumour mass within or next to mineralised chondroid tumour components [37].

In radiographs and CT scans (Figure 1, Figure 3, Figure 4 and Figure 5), in the area represented by the low–grade chondrosarcoma, there is usually (70% of cases) clear destruction, thickening and penetration of the cortex by the mass. The damage frequently originates inside the medullar cavity and spreads superficially to later perforate the cortex [7]. This may sometimes lead to cortex rupture and invasion of neighboring soft tissues [21]. Changes are described as endosteal scalloping. That is focal resorption of the cortex’s inner layer due to slow growing malignancy. The cartilage-producing part has a characteristic for chondrosarcomas rings and arc calcifications which is a process of deposition of multiple nodules of hyaline cartilage on top of one another in the shapes resembling rings and arcs [13].

In MRI, a single mass is evolving aggressively into the soft tissue around the bone. It is divided into two areas of varying signal intensities visible on T2-weighted imagining. The part producing cartilage has a high signal seen on fluid-sensitive sequences whereas the sarcomatous tumour is shown as an osteolytic area with a reduced signal intensity often intensified by contrast [38] (Figure 1, Figure 3, Figure 4 and Figure 5). However, only in one third of MRI scans and radiographs do the lesions have bimorphic features. They are visible only in half of the CT scans, so histological examination is equally necessary to diagnose the disease [39]. Areas of dedifferentiation within chondrosarcoma can be detected on T2-weighted MRI as regions with decreased signal intensity. These particular areas should be prioritised when selecting the biopsy site [40]. Ultrasonography (USG) can also detect large lesions with extraosseous extensions for biopsy [41].

#### 3.4.2. Histopathology

The most common histological criterium for diagnosing chondrosarcoma is a lack of bone formation and synthesis of hyaline cartilage [42]. To diagnose the dediffrentiated subtype, two separate components must be found after the biopsy of the mass during both macroscopic and microscopic examinations: low-grade cartilaginous tumour representing the development of chondrosarcoma and high-grade mesenchymal, frequently not differentiated and non-cartilaginous tissue evolving into a sarcoma [20]. Microscopically, there is a clear demarcation line that separates both components. Macroscopically, the former’s color pattern ranges from blue to grey and is a lobulated area in the center of the bone. In contrast, the latter is fleshy and white with visible effects of hemorrhage with an extraosseous localisation [27,43]. Histological examination also has limitations since it is easy to omit one or the other component during tissue sampling [26]. It is best to carefully plan the biopsy procedure so it should be done after an MRI [4,19].

#### 3.4.3. Image-Guided Percutaneous Core–Needle Biopsy

As mentioned before, for a biopsy to be well planned and reveal a correct pathology diagnosis, radiological imagining must be performed along with it. Currently, a procedure that allows combination of these two methods, and is also reliable, is a percutaneous core–needle biopsy guided by radiological imagining. It is becoming increasingly accepted as a procedure for the initial diagnosis of musculoskeletal tumours and is starting to replace the open surgical biopsy [44]. The second is a more invasive procedure with a greater risk of complications, and even though it is still considered a so-called “gold standard” for the diagnosis of musculoskeletal tumours, it is becoming a method that is applied only after image-guided percutaneous core–needle biopsy is inconclusive. Image-guided biopsy is advantageous because it minimises the risk of infection or bleeding and at the same time is still effective [45]. According to research, it provides conclusive and accurate results in approximately 87–89% of cases [46,47]. The base for radiological imaging is a CT scan which is favoured more than MRI because it provides a precise image of soft tissues along with bones and allows the identification of the exact location of each component (both low-grade and high-grade) of the tumour mass without being expensive, invasive, and does not interfere with metal parts in the body [48]. If the tumour is large and has an extra-osseous component, it could be guided by USG instead of CT [41].

### 3.5. Staging

According to Union for International Cancer Control (UICC), all chondrosarcomas, including DDCS, are carried out following TNM Classification for Bone Sarcomas. In this group, all bone malignancies are located in the appendicular skeleton, trunk, skull, and facial bones. There is also a separate classification of TNM for spine and pelvic bone locations. Consequently, dedifferentiated chondrosarcoma is assessed with an appropriate scale according to its localisation in the body [49]. 

## 4. Pathology

Dedifferentiated chondrosarcoma characterised by a bimorphic histomorphology, a low-grade chondrosarcoma component and a high-grade transformed chondrosarcoma (anaplastic) component with a remarkably sharp junction between the two components. In both components, tumour cells seem to originate from a single precursor, but in the anaplastic component, there are many genetic alterations [50] (Figure 1).

### 4.1. Low–Grade Component

The low-grade component is characterised by the presence of chondrocytes and includes grade I and II. In grade I cartilages are weakly to moderately cellular and hyperchromatic, without mitosis and with an abundant hyaline cartilage matrix [3]. Chondrosarcoma grade II exhibits higher cellularity, showing increased nuclear atypia and mitotic activity. The perilobular and interlobular cells are large, round, or oval-shaped cells with a high nucleus-to-cytoplasm ratio. The cells have small, often pleomorphic nuclei and a moderate eosinophilic cytoplasm [7,51]. Under the microscope, clear epithelioid cells, mixed osteoid and chondroid areas, and giant cells are seen. 

### 4.2. High-Grade Component

In high-grade components of the DDCS cartilaginous molecules are absent except for areas with characteristics similar to those of chondroblastic osteosarcoma [52]. A high-grade component is characterised by undifferentiated round or spindle-shaped mesenchymal cells with elongated single hyperchromatic nuclei and a small volume of cytoplasm. Cells may show pleomorphism, mitotic activity, atypia, cartilaginous matrix loss, and spindle-shaped cells infiltration into low-grade components. Some cartilage zones with multinuclear tumourous giant cells and hypercellular stroma permeate the bone trabeculae and spindle cell component. In addition to the chondroblastic cells, there may also be areas of dedifferentiation where the cells have oval and elongated hyperchromatic nuclei and scanty, poorly outlined cytoplasm [51]. These cells have lost some of the characteristics of chondroblasts and can resemble cells of other types such as include undifferentiated sarcomas, osteosarcomas, angiosarcomas, fibrosarcomas, rhabdomyosarcomas, leiomyosarcomas, malignant fibrous histiocytomas, or giant cell tumours [50,53]. These areas of dedifferentiation are known to be more aggressive and have a poorer prognosis. The metastases often show only the high-grade anaplastic component [54]. There is also intramembranous (direct) and endochondral (indirect) ossification. Intramembranous ossification occurs when pluripotent mesenchymal cells enter the osteoblast lineage; however, in endochondral ossification chondrocytes differentiate and are replaced by osteoblasts [55]. Areas of necrosis may be present within the high-grade component of the tumour.

### 4.3. Cellular Infiltrates 

Several studies have shown that DDCS tumours are often infiltrated by various immune cells, including macrophages and lymphocytes [7,29,56,57,58]. Malchenko et al. study of lung metastases in DDCS observed the presence of leukocyte infiltration in these metastases, which may have been involved in the metastatic spread in DDCS. The main component of the leukocyte infiltrate in the tumour microenvironment was macrophages derived from circulating monocytes [29]. The overexpression of macrophage chemoattractant in tumours and increased macrophage density have also been shown to correlate with a poor prognosis [59,60].

In addition to macrophage infiltrates, lymphocytic infiltrates are also present. In the histopathological analysis of DDCS by Gong et al., some areas had a characteristic inflammatory infiltrated with aggregates of plasma cells and lymphocytes that mimic an inflammatory myofibroblastic tumour [7]. Clinical studies suggest that chondrosarcomas behave like inflammatory tumours, with dense lymphocytes infiltrating the tumour and high expression of checkpoint inhibitor molecules such as programmed death ligand-1 (PD-L1) [61,62,63]. Similarly, in the study by Kostine et al. which analysed DDCS samples, PD-L1 expression was observed in more than 50% of the samples. It was associated with high T-cell infiltration which may suggest that PD-L1 could be used as a biomarker or predictor of response to immunotherapy [64].

### 4.4. Immunohistochemistry (IHC)

Immunohistochemistry (IHC) using specific antibodies can help distinguish chondrosarcoma from other cartilage tumours (Table 1, Figure 2). Several molecules have been reported as biomarkers in diagnosing, prognosis, and treating DDCS. One of such biomarkers is the S100 protein, expressed in many tumours, including several sarcomas [65]. Two calcium-binding sites characterise it, modulate cellular responses, and can be used as a marker for chondroid tissue origin [66]. The expression of the S100 protein was observed in many subtypes of chondrosarcomas, such as CCCS, CCS, or MCS. Compared to other chondrosarcomas, DDCS is negative for the s100 protein [67]. Another marker is p53, encoded by the *TP53* gene, located on chromosome 17 at locus 17p13.1. P53 can be overexpressed in CCCS, CCS, MCS, and DDCS [68,69].

Another marker, positive in DDCS is also the cancer testis antigen-New York esophageal squamous cell carcinoma 1 (NY-ESO-1) encoded by the CTAG1B gene. Among normal tissues, it is expressed only in the germ cells of the adult testis and undergoes atypical re-expression in many malignancies such as CCS, synovial sarcoma or melanoma [70,71].

Isocitrate dehydrogenase (IDH) is a metabolic enzyme that catalyses the oxidative decarboxylation of isocitrate and therefore plays key roles in the Krebs cycle and cellular homeostasis [72]. Advances in cancer genetics have revealed that antibodies against the mutant IDH protein have been detected in various human malignancies, including glioma [73], cholangiocarcinoma [74], acute myeloid leukemia (AML) [75], and chondrosarcoma, such as CCS and DDCS [76,77]. However, in DDCS, the p.Arg132His mutation-specific IDH1 antibody can help identify less than 20% of these tumours [78]. Mutant IDHs were not found in CCCS and MCS [54,78]. Mutations in IDH1 can be used to differentiate DDCS from undifferentiated pleomorphic sarcoma, fibrosarcoma, and osteosarcoma [79,80]. Most importantly, a differential analysis of methylation patterns revealed a decrease in the global hypermethylation typically associated with IDH1/IDH2 in conventional chondrosarcoma. At the same time, DDCS exhibited a unique methylation profile distinct from that of conventional chondrosarcoma. Genomic examination identified an overrepresentation of TP53, TERT promoter, and CDKN2A/B alterations in DDCS but copy-number alterations in DDCS was significantly lower. Integrating methylation and gene expression analysis revealed that distinctive methylation and transcriptional profiles related to IDH1/IDH2 were early events in DDCS [80,81]. SRY-box transcription factor 9 (SOX-9) is the master regulator of chondrogenesis and increases in chondrosarcoma tissue and is directly targeted by miR-145 [82]. It is positive in DDCS, but also can be expressed in OSC or CCCS, CCS, and MCS, complicating the differential diagnosis [83]. Other essential markers in DDCS are B-cell lymphoma 2 (Bcl-2), mouse double minute 2 homologue (MDM2), and PD-L1 [84,85,86,87]. DDCS also stains positive for Ki-67, cyclin D1, desmin, collagen type 1 (Col I) and p16 [7,86,87,88,89,90]. Furthermore, inactivation of polycomb repressive complex 2 (PRC2) results in trimethylation of histone 3 in lysine 27 (H3K27me3) deficiency, which is detected as a complete loss of H3K27me3 staining [91].

**Table 1:** Comparison of markers in different subtypes of chondrosarcoma based on Zając, A.E., et al. [92].

Protein/Subtype	DDCS	CCCS	CCS	MCS
S100	-	+	+	+
P53	+ *	+	+ **	+
SOX-9	+	+	+	+
Bcl-2	+	+	+	+
IDH1	+	-	+	-
NY-ESO-1	+	-	+	-
Other	CD44, Col1a1, Col2a1, cyclin D1, MDM2, Ki-67, PAI-1, PD-L1, PTHrP, Runx2	Col2a1, keratine, PTHrP, PDGF, Runx2	Brachyury, Col2a1, Cox-2, D2-40, Gal-1, MDM2, PTHrP, YKL-40	CD99, desmin, EMA, MYF4, MYOD1, NKX2.2
References	[6,60,61,63,64,65,67,68,69,70,71]	[54,70,89,93]	[65,70,78,83,94,95,96]	[70,78,89,97,98,99,100]

* p53 can be overexpressed in 59% of cases, [54]. ** Grade 2 and 3. DDCS—dedifferentiated chondrosarcoma, CCCS—clear cell chondrosarcoma, CCS—conventional chondrosarcoma, MCS—mesenchymal chondrosarcoma, SOX9-SRY—box transcription factor 9, Bcl-2—B-cell lymphoma 2, IDH1—isocitrate dehydrogenase 1, NY-ESO-1—New York esophageal squamous cell carcinoma-1, Col1a1—collagen type II alpha 1 chain, MDM2—mouse double minute 2 homolog, PAI-1—plasminogen activator inhibitor 1, PD-L1—programmed cell death receptor ligand 1, PTHrP—parathyroid hormone-related protein, Runx2—runt-related transcription factor 2, PDGF—platelet-derived growth factor, Cox-2—cyclooxygenase-2, D2-40—podoplanin, Gal-1—galectin-1, YKL-40—chitinase 3-like 1, EMA—equi merozoite antygen, MYF4—myogenin, MYOD1—myoblast determination protein 1, NKX2.2—NK2 homeobox 2.

### 4.5. Differential Diagnosis

First, DDCS is often misdiagnosed as conventional chondrosarcoma because it is easy to omit the dedifferentiated component during biopsy (Table 1 and Table 2). Similarly, only the high-grade component could be present in the smears and then be misdiagnosed as the sarcoma representing the undifferentiated component. It could be an OSC, FS, MFH, RMS, LMS or any other previously described sarcoma that could represent the high-grade tumour. In this case, the best prediction of appearance of dedifferentiated chondrosarcoma is a medical history of enchondromas or chondrosarcomas [101].

Occasionally, the high-grade component can have a resemblance to a benign tumour. It can mimic a giant-cell tumour of bone which even though is an aggressive malignancy occurring near joints, it is also benign, non-cancerous and usually has a low-grade. It is most often malignant fibrous histiocytoma instead and the resemblance is due to large, circular cells similar to osteoclast without any identifiable anaplasia. However, what indicates that it is a malignant tumour is that there are large areas of mononuclear cells, and they perforate and damage the cortex. These are not characteristic features of giant cell tumours but, as was said, a fibrous histiocytoma instead [5,102].

**Table 2:** Immunohistochemical differentiation in different types of sarcoma.

References	Type of Sarcoma	S100	P53	MDM2	Ki-67	Desmin	SMA	EMA	Vimentin	Myosin	h-Caldesmon	Others
[7,85,86,88,89,90,103]	DDCS	-	+	+	+	-	-	-	±	±	±	CD99
[104,105,106,107,108,109]	OSC	+	+	+	+	±	±	-	+	-	-	CD10, CD99, PAX2
[104,110,111,112,113,114]	FS	+	-	+	+	+	+	+	+	+	+	Cd34, CD99
[115,116,117,118]	MFH	-	±	±	±	+	+	±	+	±	-	Cd45, CD68
[104,110,119,120,121,122]	RMS	-	-	±	+	+	-	+	+	+	±	Myogenin, CDK4, CD56, CD99
[104,110,115,119,123,124]	LMS	-	-	-	±	+	+	±	±	+	+	Calponin, CDK4, CD34

± negative or weakly positive in some cases. MDM2—mouse double minute 2 homolog, SMA—smooth muscle actin, EMA—epithelial membrane antigen, DDCS—dedifferentiated chondrosarcoma, OSC—osteosarcoma, FS—fibrosarcoma, MFH—malignant fibrous histiocytoma, RMS—rhabdomyosarcoma, LMS—leiomyosarcoma, PAX2—paited box gene 2.

## 5. Genetics

Until now, we have insufficient data on molecular abnormalities in chondrosarcomas. The knowledge of the molecular basis of chondrosarcomas is extremely important for understanding the pathogenesis of these tumours and their specific subtypes. Numerous studies are being conducted on using specific mutations as potential targets in new therapies and prognostic factors [125,126,127,128].

### 5.1. IDH Mutations

One of the most common mutations observed in many cases of chondrosarcoma, including DDCS, is mutations in the *IDH1* and *IDH2* genes. Mutation in *IDH1*/*2* leads to the production of 2-hydroxyglutarate (2-HG), an oncometabolite that contributes to epigenetic changes such as histone methylation and DNA aberration [129]. Research conducted by Makoto Nakagawa et al. revealed that 2-HG is a potentially significant biomarker for the presence of *IDH* mutations [130]. *IDH* mutations are more common in high-grade subtypes of chondrosarcoma, including DDCS [78,125,126,127]. In the study by Amary et al., 13 of 23 (56.5%) cases of DDCS harbored *IDH* mutation (12 cases in *IDH1* R132C/G/H/L and 1 in *IDH2* R172S), and in central low-grade cartilaginous tumours IDH mutations were observed in 52% of cases [78]. Another study found that IDH1/IDH2 mutations were observed in 9/14 (64.3%) DDCS cases. Furthermore, in the study by Nakagawa et al. *IDH2* mutations were observed in 5/6 (about 80%) DDCS [126]. The IDH mutation can be found in conventional and dedifferentiated components used in diagnostic molecular pathology [131]. Research conducted by William Cross et al. revealed an association between *IDH2* mutations and an increased frequency of mutations in the *TERT* promoter in chondrosarcoma; however, such a connection was not present among DDCS [132]. However, mutations in the *TERT* promoter were observed in approximately 56% of 63 DDCS cases.

Some studies revealed that the presence of *IDH1*/*2* mutations was associated with a worse prognosis in central chondrosarcoma [125,126,127]. Similar results were also observed in DDCS with *IDH2* mutations, resulting in worse metastasis-free survival and overall survival (OS) [126]. However, the association between *IDH* mutations and overall survival may be associated with tumour grade and the common occurrence of these mutations in high-grade tumours [127].

*IDH* mutations may be useful in the differentiation of neoplastic lesions. Their presence is observed in cartilaginous tumours, including chondrosarcomas (besides clear cell and mesenchymal subtypes), whereas these are absent in mesenchymal tumours, such as osteosarcoma or undifferentiated pleomorphic tumours (UPS) [127]. The study conducted by Chen et al. demonstrated that *IDH* mutations were present in 87% (20/23) of DDCS and no *IDH* mutations were identified among 14 patients with UPS [79]. *IDH* mutation may help distinguish chondrosarcoma from osteosarcoma, which is crucial in treatment selection and response. Neoadjuvant chemotherapy is an established method of treatment of osteosarcoma [127].

### 5.2. TP53 Mutations

Another frequent mutation in DDCS refers to the *TP53* gene. In general, mutations in this gene are the most common among all tumours. *TP53* mutations were detected in 20–50% CCS and DDCS [133]. *TP53* acts as a tumour suppressor gene; therefore, the loss of its function leads to the development of carcinogenesis [134].

The study by Sandberg et al. indicated that loss of heterozygosity (LOH) in *TP53* may contribute to the transformation of pre-existing low-grade conventional chondrosarcoma into a highly malignant dedifferentiated tumour and, contrary to *IDH* mutations, is considered to be a late event in the development of DDCS [135,136]. Furthermore, the presence of *TP53* mutations was associated with increased malignancy of chondrosarcomas [133,137,138]. In a study by Yang Li et al., conducted in a mouse model, double deletion of the *TP53* and *RB1* genes in chondrocytes resulted in increased activity of the YAP pathway, contributing to the induction of chondrosarcoma. These researchers also demonstrated metformin’s inhibitory effect on the YAP pathway, paving a new potential path for chondrosarcoma therapy [128].

### 5.3. Other Mutations

Other mutated genes in DDCS include the cyclin-dependent kinase inhibitor 2A (*CDKN2A*)/cyclin-dependent kinase inhibitor 2B (*CDKN2B*) related to the Rb pathway [54]. Both genes are located in chromosome 9 and encode p16 and p14/p15 proteins respectively [139]. In the study by Tarpey et al., homozygous deletion of *CDKN2A* was found in up to 36% (5 of 14 cases) of DDCS [140]. In another study, 62% (13 of 21 cases) of DDCS cases had *p16/CDKN2A* loss, whereas polysomy in this chromosome region was observed in 28.6% (6 of 21 cases) of DDCS. Moreover, in three cases, p16/CDKN2A loss was confirmed to be present in the dedifferentiated component and not in the well-differentiated component [141]. On the other hand, in a study by Meijer et al., loss of p16 expression was observed in both anaplastic components (79%) and the cartilaginous components (82%) of DDCS [54].

*COL2A1* gene, which encodes the alpha chain of type 2 collagen, has also been observed in DDCS and are likely early events in progression of these tumours [92,136,140]. In the study by Tarpey et al., 35.7% (5 of 14 cases) of DDCS harbored missense or insertion/deletion mutations. The dysfunction of *COL2A1* may result in abnormalities in matrix deposition and signal transduction pathways [140,142], leading to abnormal and uncontrolled divisions and tumour development [132].

Other identified mutations in DDCs occurred in genes related to the Hedgehog pathway, i.e., hedgehog-interacting protein (*HHIP*) (2/14 cases of DDCS), glioma-associated oncogene homolog 1 (*GLI 1*) (1/14), and protein-patched homolog 1 (*PTCH1*) (1/14) [140]. In isolated cases of DDCS, mutations were identified in tuberous sclerosis 1 (*TSC1*), neurofibromatosis type 2 (*NF2*), lysine demethylase 6A (*KDM6A*) [140] and homolog phosphatase and tensin (*PTEN*), janus kinase (*JAK*), neurotrophic tyrosine receptor kinase (*NTRK*), neurogenic locus notch homolog protein (*NOTCH*), and mitogen-activated protein kinase (*MAPK*) [143] genes were identified.

Moreover, some chromosomal abnormalities were detected in DDCS. One of such examples is the trisomy of chromosome 19, detected in more than 50% of DDCS [50,144]. Among other chromosomal aberrations, tetrasomy of chromosome 7 was observed in two cases [144], and aberration of the long arm of chromosome 5 [50]. Furthermore, it is considered that 5q14.2-q21.3, 6q16-q25.3, 9p24.2-q12, and 9p21.3 are characteristic for DDCS [90].

## 6. Treatment

### 6.1. Surgical Treatment

Despite the lack of a precise treatment protocol, surgery remains the standard of care for patients (Figure 3), and it is shown to be successful primarily in patients whose tumour has not metastasised [145]. Due to the presence of the high–grade component, there is a high risk of metastasis and local recurrence even after resection; therefore, an achievement of a wide or radical surgical margin is required [146]. The chance of recurrence varies in the literature and ranges from 18% to 45% [147]. However, Stevenson et al. found that the greater the margin, the less likely was LR, and the most effective margin was greater than 4 cm stretching from the tumour [146]. There is no proof that additional chemotherapy accompanying surgical treatment makes it more successful [148]. According to Mercury et al., no therapy is sufficient to prevent death from the disease, which is most often caused by lung metastasis [4,148].

### 6.2. Treatment of Localised Disease

Surgery is the most effective treatment for localised DDCS, improving survival rate after amputation or surgery with limb salvage. Wide margins correlate with a longer period of survival [16]. Local recurrence of DDCS is linked to inadequate margins of excision; the presence of pathological fractures showed relevance in impacting LR in previous studies, yet a recent study suggests a lack of substantial influence of pathological fracture on the prognosis of patients with DDCS [149]. An initial resection reduces the chance of local recurrences. Tumours are considered resectable as long as there is a possibility to improve the patient’s condition and if the location and tumour size allow for resection.

Pathological fractures in chondrosarcoma are accompanied by peritumoural edema, cortical disruption, and the calcification and endosteal scalloping in the cartilaginous portion of the tumour. Treatment in such cases is radical surgery [150,151]. In patients with a large localised disease and a pathological fracture in long bones, an amputation might be beneficial in reducing the risk of LR. Reconstruction might be considered in the case of a successful local control achieved with limb salvage [152]. Other treatment options for localised disease include radiotherapy (RT). Overall, histological subtype, tumour size, and chemotherapy did not show significant evidence of affecting LR or survival [10].

### 6.3. Ratiotherapy

The role of radiotherapy remains controversial as some authors support its importance and others question it [153]. Chondrosarcomas are relatively resistant to RT [154]. RT can be considered in two cases: after resection, aiming for maximum local control, and when resection is impossible, such as palliative treatment (Figure 4) [3]. Treatment of choice is a combination treatment consisting of complete surgical resection of the tumour with a maximal excision and then adjuvant radiotherapy. The treatment method used is high-dose radiotherapy (>60 Gy) with proton beam radiation and radiosurgery. It provides better local control and increased survival [154,155].

Retrospective studies have shown longer time free of local progression following adjuvant radiotherapy and radical radiotherapy resulted in tumour regression. For grade I and II, adjuvant radiotherapy are not indicated [155,156]. However, a study by Krochak et al. showed that patients with low-grade malignancy had more favourable results concerning local control than those with higher grades [157]. However, some studies have demonstrated apart from surgical treatment, there is no consensus on the role of adjuvant radiation in DDCS. Use of any adjuvant treatment has no survival benefit and is likely in palliative medicine [9]. It can be concluded that radiotherapy does not impact overall or specific survival. There may be consideration if DDCS is not subject to complete surgical resection or if it should be treated with palliative treatment [17,20,21].

Doses of RT in palliative treatment ranged from 30 to 70 Gy, depending on the site and size of the tumour (Figure 5). There are data that patients did not achieve significant long-term benefit, but it is also possible in some cases [156,158].

### 6.4. Treatment of Metastatic Disease

While metastases develop quickly in DDCS, the treatment options in metastatic disease are limited. The success of chemotherapy and radiotherapy remain debatable with varying results. Metastases develop over a few months from diagnosis in 90% of patients, a common location being lungs [29,159]. Metastases at the diagnosis are a prevalent negative prognostic factor showing a significantly low OS (10% of survival at 2 years, median survival of 5 months) [10]. In a study of 23 patients, 8 presented with lung metastases at diagnosis [18]. The influence of metastases on survival is the most significant in DDCS among all non-conventional chondrosarcoma subtypes (19,8% of marked difference in presence of metastasis). Among the affected patients, the additional poor prognostic factors were pelvic location of the tumour and increasing age [8].

### 6.5. Palliative Treatment

DDCS is a rare and aggressive type of bone cancer that often requires multimodal treatment approaches to manage its progression [3,9,154]. While surgical resection and chemotherapy are the primary curative treatments, some cases may present with metastasis or recurrence that is unresponsive to conventional therapies [145,159]. In these situations, the focus of treatment shifts towards palliative treatment. Palliative treatments for dedifferentiated chondrosarcoma may involve chemotherapy [10,18], immunotherapy [58,61,64], and targeted treatment [160,161].

#### 6.5.1. Chemotherapy

Chondrosarcomas, in general, are resistant to chemotherapy [162]. Short-term local control can occasionally be achieved but has no proven benefit on distant spread or overall survival [158]. The role of chemotherapy is controversial in terms of impact on overall survival [10,18,43,163,164]. The first-line treatment regimens used for localised and metastatic dedifferentiated DDCS follow protocols used for osteosarcoma [165,166] (Table 3).

In retrospective study by Lex et al., patients with DDCS of the pelvis were enrolled. Patients received surgical treatment and palliative chemotherapy using cisplatin (CP) and doxorubicin (DOX). In patients who received chemotherapy, OS at 12 months was 15.4% and 55.6% for those treated with surgery [158]. The presented results are very similar to those of Maldegem et al. In their study, the researchers published results on treatment in unresectable DDCS, which demonstrate that patients treated with DOX monotherapy had progression-free survival (PFS) of 5.5 months and patients treated with a combination of doxorubicin, cisplatin, and methotrexate (MTX) had a PFS of 2.9 months [161]. In a study of a European network, a wide range of chemotherapy drug regimens were used for DDCS. The most common was the combination of DOX and CP and DOX and ifosfamide (IF). The 5-year survival in patients treated by chemotherapy was 33% and in patients who were not was 25%. Results from a European group study on DDCS revealed that patients with metastatic disease have a particularly unfavourable prognosis. The efficacy of palliative interventions such as chemotherapy in significantly enhancing the outcome has not been demonstrated to significantly improve outcome [10].

In a report of nine cases in patients with DDCS, chemotherapy was given to four patients after surgical tumour resection. The chemotherapy consisted of adriamycin, ifosfamide, cisplatin, and methotrexate. There were no significant differences in survival between patients who did or did not receive chemotherapy. However, the median OS in cases where chemotherapy was received and was not received was 11.8 vs. 9.1 months, respectively [19]. According to Italiano et al., chemotherapy has limited efficacy in patients with advanced chondrosarcoma. In their study, patients received various combinations of DOX, CP, and IF. The highest benefit is observed in DDCS than in other chondrosarcomas. The overall response survival (ORR) for DDCS was 20.5%. Combination chemotherapy was associated with a higher ORR and PFS, but did not improve OS [167]. Analysis of the SEER database of DDCS patients also showed that chemotherapy treatment was not associated with improved OS and should only be used after careful consideration [168].

However, a retrospective study conducted by Mitchell et al. investigated the treatment of DDCS, and found that patients received a combination of DOX and CP. Among these patients, the 5-year survival rate was 36%. It was observed that patients who received chemotherapy had more favourable outcomes than those who did not receive chemotherapy or were treated with surgery alone. Notably, the number of patients in each group was very small [169]. Also, in a study by Kawaguchi et al. of ifosfamide therapy and chemotherapy in DDCS, chemotherapy was administered with surgery or in combination with radiation therapy and pre- or postoperatively. The treatment regimens used included a combination of DOX, CP, and IF. Cytostatic doses were 60–75 mg/m^2^ DOX, 100–120 mg/m^2^ CP and 10 g/m^2^ IF in three to four preoperative and one to nine post-operative cycles. The median disease-specific survival time was 18 months. The survival rates were 33% and 15%. Patients treated with IF had 2- and 5-year survival rates of 54% and 27%, and patients not treated with IF had 2- and 5-year survival rates of 17% and 6%, respectively. This study reports an apparent benefit of chemotherapy on survival in patients with DDCS. However, ifosfamide therapy caused acute renal toxicity and encephalopathy [165]. In addition, the 2021 study by Hompland et al. also noted renal toxicity and neurotoxicity, but also that chemotherapy could be considered in patients with DDCS. They administered 60 mg/m^2^ DOX, 100 mg/m^2^ CP, 6 g/m^2^ IF, and 8 g/m^2^ MTX to patients with poor histologic response. Patients received chemotherapy in nine cycles if it was neoadjuvant treatment, three cycles for primary chemotherapy, and six cycles for postoperative chemotherapy. The median OS was 24 months, and the 5-year survival was 39%. Patients older than 40 years had the best outcomes [166]. The van Maldegem et al. study, which used chemotherapeutic treatment in DDCS, showed that doxorubicin monotherapy appears to have an unexplained better PFS than combination therapy based on doxorubicin with cisplatin and methotrexate (5.5 vs. 2.8 months) [168]. In the most recent study, 36 patients underwent systemic therapy, 13 receiving therapy in the neo/adjuvant setting and 30 receiving therapy for metastatic disease, with 7 receiving chemotherapy in both settings. The most frequently administered regimen was doxorubicin and cisplatin (AP), sometimes with methotrexate (n = 15). Gemcitabine/docetaxel (GD) (n = 10) was also commonly used in the metastatic setting. The overall response rate to systemic therapy was 9% (n = 4), with no complete responses observed. Among the respondents, three were treated with an anthracycline-based regimen, including two patients who received a combination of doxorubicin and ifosfamide (AI) and one receiving doxorubicin and cisplatin (AP). One patient received single-agent pembrolizumab. Most patients experienced disease progression as the best response to treatment (55%, n = 26) [162].

DDCS remains a major therapeutic challenge. When comparing studies examining chemotherapy’s efficacy in DDCS, treatment remains controversial and generally does not significantly impact overall survival. However, recent studies have shown that chemotherapy can be considered for treating patients older than 40 [20,21,159,166,168,170].

#### 6.5.2. Immunotherapy

In addition to various palliative treatment options, there is growing clinical evidence suggesting the potential of immunotherapy as a viable option for patients with advanced DDCS. However, the studies investigating the effectiveness of immunotherapy in advanced DDCS are limited.

In a case report study by Singh et al., which examined the effect of immunotherapy on PD-L1-positive DDCS, pembrolizumab (200 mg every 3 weeks) was administered after palliative resection. The patient showed a remarkable response with regression of metastatic foci and a complete sustained response for 24 months [58]. Among 22 whole-tissue samples of DDCS analysed by Kostine et. al., PD-L1 expression was seen in 52% of samples and was associated with high T-cell infiltration. The median OS was 10 months for patients with PD-L1 positive DDCS and 19 months for patients with PD-L1 negative tumour, although PD-L1 expression did not significantly correlate with overall survival [64]. The study by Iseulys et al. in 49 tumour samples showed PD-L1 positivity in 42% of the patients. They have found that tumour-associated macrophages were the dominant type of immune cell in the immune environment of chondrosarcoma [171]. Areas of high lymphocyte density and PD-L1 expression were correlated with dedifferentiated parts of the tumour. This suggests that immunotherapy could be directed at this component of tumours, which is usually resistant to chemotherapy [172]. According to Tawbi et al., 95% of patients had a progression event after receiving 200 mg of pembrolizumab every 3 weeks. The median PFS was 8 weeks; the median OS was 52 weeks [61]. Wagner et al. published results of 67-year-old man treated with intravenous injection (iv) of nivolumab 240 mg every 2 weeks. The study demonstrated a partial response after four cycles of nivolumab. The tumour was positive for PD-L1 as PD-L1 expression was identified [173]. Also, in the study by Paoluzzi et al. patients were treated with the PD-L1 inhibitor, nivolumab. The studies demonstrated a partial response after six cycles of nivolumab with only 6% ORR. Interestingly, one patient with DDCS observed a partial response and a higher PD-L1 expression than all other tested patients (20% vs. less than 5%) [174].

One of the interesting studies is the ImmunoSarc phase ½ study of sunitinib (SU) and/or nivolumab (NI) plus chemotherapy in advanced soft tissue and bone sarcomas. One of the eight cohorts is for patients with DDCS. Their main objective is to assess the combination of efficacy of the sunitinib plus nivolumab measured by progression-free survival rate (PFSR) at 6 months and secondary endpoints OS and ORR. 37.5 mg/day SU i.v. was given in days 1–14 and then reduced to 25 mg/day and 3 mg/Kg NI was given every 2 weeks from week 3. The cohort results show one patient with DDCS lasting 22 months and ongoing (complete response 2.5%) [175].

#### 6.5.3. Targeted treatment

Multiple preclinical studies suggest that cell proliferation and survival pathways may be potential therapeutic targets for treating DDCS, using tyrosine-kinase inhibitors (TKI) or IDH inhibitors or monoclonal antibodies (MoAb). A preclinical study by Zhang et. al. showed that in chondrosarcoma cells, multiple tyrosine-kinase receptors (TKR) are highly activated and have a crucial role in mediating DDCS cells growth. These authors explored the effect of targeting the common TKR signaling pathways. They found that the dual pan-class I inhibitor phosphoinositide 3-kinase (PI3K)/mTOR BEZ235 significantly inhibits chondrosarcomas’ growth in vivo and in vitro. In 44% of the clinical samples, strong phosphorylation of S6 kinase was detected, and a surrogate of the activity of the phosphoinositide-3 kinase/mammalian target of rapamycin (PI3K/mTOR) pathway activity was detected after treatment with TKI. This suggests that TKRs are important mediators of chondrosarcoma cell growth and may be a promising target for future therapies [176]. Increased activation of the PI3K/mTOR pathway is also often associated with resistance to cytotoxic therapies, which make it a promising target for future pharmacological interventions [160]. Sadly, in most triasl of mTOR inhibitors including the everolimuas NCT02008019 CHONRAD trial NCT02008019, mesenchymal, dedifferentiated, clear cell subtype chondrosarcoma, and soft tissue chondrosarcoma are excluded from the study. In a study conducted on chondrosarcoma cell lines, Polychronidou et al. demonstrated that the constitutive activation of PI3K/mTOR may be mediated by the overexpression of platelet-derived growth receptor (PDGFR) and insulin-like growth factor 1 receptor (IGF-R1) [177].

Furthermore, chondrosarcoma kinome analysis showed constitutive activation of PI3K/mTOR, PDGFR and Src pathways [178]. A retrospective study by Molho et al. of unresectable chondrosarcoma (10% of total was DDCS), who were treated with the mTOR inhibitor sirolimus in combination with cyclophosphamide, showed that the disease control rate was 70%, the median PFS was 13.4 months, and the median OS was 15.5 months [179]. Currently torisel and liposomal doxorubicin are tested in patients with advanced soft tissue and bone sarcomas in Sidney Kimmel Comprehensive Cancer Center at Johns Hopkins University in a NCT00949325 trial. In another mTOR trial, sapanisertib was tested only in xenografts and cell lines [180].

Unlike mTOR studies the Italian Sarcoma Group in the phase II trial of imatinib in patients with nonresectable high-grade chondrosarcomas did not show significant clinical activity, even though imatinib was well tolerated [181]. Additionally, in study by Schrage et al., using kinome profiling in primary chondrosarcoma cultures, imatinib did not show any effect on chondrosarcoma cells. In contrast, dasatinib may provide a potential therapeutic benefit for chondrosarcoma patients who are not qualified for surgery, because a decrease in cell viability at nanomolar concentrations was found in seven of nine chondrosarcoma cultures [178]. In the phase II study of patients with CS (almost 31% of the trial) with dasatinib administration (dasatinib was given 100 mg orally once a day and the treatment cycle was 28 days) in Choi Response Criteria ORR was 15%, 6-month PFS was 47%, median PFS was 5.5 months, 2-year OS was 56%, and 5-year OS was 9%. In RECIST Response Criteria ORR of CS is 0% [182]. Preliminary clinical data from dasatinib treatment in patients with chondrosarcoma have shown modest efficacy and support further study of dasatinib in CS [182,183].

IDH-targeted therapies trials also recruited patients with dedifferentiated chondrosarcoma. Six patients had a dedifferentiated histology in a trial with Mutant IDH1 Inhibitor Ivosidenib. In this trial, the median progression-free survival (PFS) duration was 5.6 months, with a 95% confidence interval ranging from 1.9 to 7.4 months. The PFS rate at the 6-month mark was 39.5%. Of the 21 patients, 11 individuals (52%) achieved stable disease during the study [77]. There are multiple other IDH-targeted therapies in development [184]. In a phase II trial, the compound AG-120 is tested in patients with chondrosarcoma mutant IDH1. At the same time, the same time phase I trial of oral LY3410738 is also ongoing in patients with advanced solid tumours mutant with isocitrate dehydrogenase 1 (IDH1) arginine 132 (R132), including but not limited to cholangiocarcinoma, chondrosarcoma and glioma or isocitrate dehydrogenase 2 (IDH2) arginine 140 (R140) or arginine 172 (R172) mutant cholangiocarcinoma. For these trails, no data is yet available. In a trial, NCT02273739 AG-221 (IDH2 inhibitor) and NCT02073994 trial AG-120 (IDH1 inhibitor) are also tested [181].

In addition to the signaling pathways mentioned, chondrosarcoma also exhibits strong activation of the Hedgehog pathway and GDC-0449 (Hedgehog inhibitor) is of interest in chondrosarcoma [180]. The French Sarcoma Group, in phase II of a collaborative study of antagonists of the Hedgehog signal pathway vismodegib (GDC-0449) in patients with advanced CS (11% DDCS), administered 150 mg/day GDC-0449 in a 28-day cycle. In RECIST, Response Criteria ORR was 0%, 6months PFS was 28%, and median PFS was 3.5 months. The median OS was 12 months. Although there are Hedgehog pathway abnormalities in DDCS, the applications in this study of vismodegib did not reach the primary endpoint [185]. The latest study of signaling pathways, involving an enhancer of zeste 2/human sulfatase (EZH2/SULF1) axis in mice, demonstrated that the EZH2/SULF1 axis mediates cMET pathway in chondrosarcoma. The researchers showed that cMET inhibitors, such as crizotinib, provide a therapeutic potential for further development as a targeted therapy in chondrosarcoma [186].

## 7. Clinical Trials

There are no clinical trials specifically dedicated to DDCS. In general, early-phase clinical studies allow enrolling patients with different tumour types, including DDCS, to assess the safety and the preliminary signs of efficacy [39,180]. Few trials enrol only patients with sarcoma, especially with the dedifferentiated mesenchymal subtype. An interesting study assessing a new strategy is the trial of LN-145 or LN-145-S1 in treating patients with relapsed or refractory ovarian cancer, triple-negative breast cancer (TNBC), anaplastic thyroid cancer, soft tissue sarcomas, osteosarcoma, or another bone sarcoma including DDCS (NCT03449108) [187]. This phase 2 study assesses how well autologous tumour-infiltrating lymphocytes LN-145-S1 work in patients with refractory DDCS. The participants receive LN-145-S1 in combination with immunotherapy (nivolumab and ipilimumab). TILs LN-145-S1 are autologous tumour-infiltrating lymphocytes (TILs) isolated from an autologous tumour sample and expanded *ex vivo* in the presence of interleukin-2 (IL-2). The LN-145-S1 TILs specifically recognize, target, and kill patient tumour cells [188]. The efficacy is assessed using RECIST 1.1 and the primary end-point is ORR. Secondary endpoints include DCR, DOR, PFS, OS, and the safety profile of adoptive cell therapy with tumour-infiltrating lymphocytes (TIL) in solid tumours. According to the NCCN 2023 guidelines in patients with metastatic chondrosarcoma, participation in any type of clinical trial participation is a valuable option [189]. Patients with dedifferentiated chondrosarcoma are eligible for the NCT02821507 trial of Sirolimus and cyclophosphamide in metastatic or unresectable myxoid liposarcoma and chondrosarcoma, and in the NCT01267955 trial of Vismodegib to treat patients with advanced chondrosarcomas. Patients with cholerasarcoma are also recruited in the NCT02389244 trial, a Phase II Study Evaluating Efficacy and safety of Regorafenib in Patients With Metastatic Bone Sarcomas (REGOBONE) [190]. regorafenib Other trials that recruit all sarcoma subtypes are the trial of Sunitinib and/or Nivolumab Plus Chemotherapy in Advanced Soft Tissue and bone sarcomas with sunitinib/nivolumab, epirubicin, ifosfamide, doxorubicin, dacarbazine, cisplatin, and methotrexate, as well NCT03670069 trial with JAK-1 inhibitor Itacitinib in the treatment of patients with refractory metastatic/advanced sarcomas; NCT03449108 trial of LN-145 or LN-145-S1 in the treatment of patients with relapsed or refractory ovarian cancer, anaplastic thyroid cancer, osteosarcoma, or other bone and soft tissue sarcomas [39].

## 8. Conclusions

DDCS has a poor 5-year survival rate (7–24%); it is difficult to diagnose and no standard treatment is available. Diagnosis is based on histological and radiological methods, but they have limitations because they do not always show the dual nature of the tumour The most common mutation present in DDCS is a mutation in the isocitrate dehydrogenase (IDH) genes present in almost 60% of lesions. It is a suggested DDCS marker. In addition, a more aggressive course of the disease is associated with increased gene methylation in cancer cells. Standard localised treatment is based on surgical resection. There are no established guidelines for the treatment of advanced-stage patients. Wide surgical margins are preferred because they correlate with higher survival rates and reduced risk of recurrence. Palliative treatment is based on immunotherapy and chemotherapy, to which DDCS is relatively resistant. Often, this characteristic is associated with increased activation of the PI3K/mTOR pathway, which provides hope for targeted therapy with inhibitors of the PI3K/mTOR pathway such as sirolimus.

Considering the above, it is important to continue research on DDCS to improve diagnosis, find more effective therapies, and prevent metastasis. Moreover, DDCS is also a rare cancer type, so a compendium of knowledge about it is necessary to allow rapid diagnosis and facilitate the selection of an appropriate treatment type.

## Figures and Tables

**Table 3:** Chemotherapy regimens used in patients with dedifferentiated chondrosarcoma.

Cytostatics	Dosing	Cycles	Reference
DOX	60–75 mg/m^2^	PRC—3–4 cycles POC-1 9 cycles **	[166]
CP	100–120 mg/m^2^		
IF	10 g/m^2^		
DOX	60 mg/m^2^ (24 h iv infusion)	AC—9 cycles	[165]
CP	100 mg/m^2^ (48–72 h of iv infusion)	PC—3 cycles	
IF	6 g/m^2^ (3 g/m^2^ per day, 1–2 h i.v. infusion)	POC—6 or 11 cycles ***	
MTX *	8 g/m^2^ (4 h i.v. infusion)		

* For patients with poor histologic response after surgery. ** Most patients undergoing 3 cycles or more. *** 6 cycles for good histological responders or 11 cycles including 5 cycles of methotrexate for patients with poor response. DOX—doxorubicin, CP—cisplatin, IF—ifosfamide, MTX—methotrexate, iv—intravenous, PRC—preoperative chemotherapy, POC—postoperative chemotherapy, AC—adjuvant chemotherapy, PC—primary chemotherapy.

## Data Availability

Not applicable.

## References

[1] Reith J.D., Bauer T.W., Fischler D.F., Joyce M.J., Marks K.E. (1996). Dedifferentiated chondrosarcoma with rhabdomyosarcomatous differentiation. Am. J. Surg. Pathol..

[2] Wittig J.C. Dedifferentiated Chondrosarcoma. https://tumorsurgery.org/tumor-education/bone-tumors/types-of-bone-tumors/dedifferentiated-chondrosarcoma.aspx?fbclid=IwAR08vgOA3CET_NS6UJaczgQK98xoKICoQKeFhBWD6VlXo6XKZgPGdxj_2XI.

[3] Gelderblom H., Hogendoorn P.C., Dijkstra S.D., van Rijswijk C.S., Krol A.D., Taminiau A.H., Bovée J.V. (2008). The clinical approach towards chondrosarcoma. Oncologist.

[4] Mercuri M., Picci P., Campanacci L., Rulli E. (1995). Dedifferentiated chondrosarcoma. Skelet. Radiol..

[5] Estrada E.G., Ayala A.G., Lewis V., Czerniak B. (2002). Dedifferentiated chondrosarcoma with a noncartilaginous component mimicking a conventional giant cell tumor of bone. Ann. Diagn. Pathol..

[6] Rozeman L.B., de Bruijn I.H.B., Bacchini P., Staals E.L., Bertoni F., Bovée J.V.M.G., Hogendoorn P.C.W. (2009). Dedifferentiated peripheral chondrosarcomas: Regulation of EXT-downstream molecules and differentiation-related genes. Mod. Pathol..

[7] Gong L.H., Su Y.B., Zhang W., Liu W.F., Dong R.F., Sun X.Q., Zhang M., Ding Y. (2021). Dedifferentiated Central Chondrosarcoma: A Clinical, Histopathological, and Immunohistochemical Analysis of 57 Cases. Front. Med..

[8] Amer K.M., Munn M., Congiusta D., Abraham J.A., Basu Mallick A. (2020). Survival and Prognosis of Chondrosarcoma Subtypes: SEER Database Analysis. J. Orthop. Res..

[9] Gusho C.A., Lee L., Zavras A., Seikel Z., Miller I., Colman M.W., Gitelis S., Blank A.T. (2022). Dedifferentiated Chondrosarcoma: A Case Series and Review of the Literature. Orthop. Rev..

[10] Grimer R.J., Gosheger G., Taminiau A., Biau D., Matejovsky Z., Kollender Y., San-Julian M., Gherlinzoni F., Ferrari C. (2007). Dedifferentiated chondrosarcoma: Prognostic factors and outcome from a European group. Eur. J. Cancer.

[11] van Praag Veroniek V.M., Rueten-Budde A.J., Ho V., Dijkstra P.D.S., Fiocco M., van de Sande M.A.J. (2018). Incidence, outcomes and prognostic factors during 25 years of treatment of chondrosarcomas. Surg. Oncol..

[12] Hua K.C., Hu Y.C. (2020). Treatment method and prognostic factors of chondrosarcoma: Based on Surveillance, Epidemiology, and End Results (SEER) database. Transl. Cancer Res..

[13] Gaillard F.J., Kusel K. Dedifferentiated Chondrosarcoma. https://radiopaedia.org/articles/6250.

[14] Alqubaisi A., Oliveira I., Singla N., Chavda A., Khoo M., Saifuddin A. (2021). The incidence and diagnostic relevance of pathological fracture in conventional central chondrosarcoma. Skelet. Radiol..

[15] Saifuddin A., Mann B.S., Mahroof S., Pringle J.A., Briggs T.W., Cannon S.R. (2004). Dedifferentiated chondrosarcoma: Use of MRI to guide needle biopsy. Clin. Radiol..

[16] Miao R., Choy E., Raskin K.A., Schwab J.H., Nielsen G.P., Deshpande V., Chebib I., DeLaney T.F., Hornicek F.J., Cote G.M. (2019). Prognostic Factors in Dedifferentiated Chondrosarcoma: A Retrospective Analysis of a Large Series Treated at a Single Institution. Sarcoma.

[17] Strotman P.K., Reif T.J., Kliethermes S.A., Sandhu J.K., Nystrom L.M. (2017). Dedifferentiated chondrosarcoma: A survival analysis of 159 cases from the SEER database (2001–2011). J. Surg. Oncol..

[18] Liu C., Xi Y., Li M., Jiao Q., Zhang H., Yang Q., Yao W. (2017). Dedifferentiated chondrosarcoma: Radiological features, prognostic factors and survival statistics in 23 patients. PLoS ONE.

[19] Yokota K., Sakamoto A., Matsumoto Y., Matsuda S., Harimaya K., Oda Y., Iwamoto Y. (2012). Clinical outcome for patients with dedifferentiated chondrosarcoma: A report of 9 cases at a single institute. J. Orthop. Surg. Res..

[20] Staals E.L., Bacchini P., Bertoni F. (2006). Dedifferentiated central chondrosarcoma. Cancer.

[21] Johnson S., Têtu B., Ayala A.G., Chawla S.P. (1986). Chondrosarcoma with additional mesenchymal component (dedifferentiated chondrosarcoma). I. A clinicopathologic study of 26 cases. Cancer.

[22] El Abiad J.M., Robbins S.M., Cohen B., Levin A.S., Valle D.L., Morris C.D., de Macena Sobreira N.L. (2020). Natural history of Ollier disease and Maffucci syndrome: Patient survey and review of clinical literature. Am. J. Med. Genet. A.

[23] Schwartz H.S., Zimmerman N.B., Simon M.A., Wroble R.R., Millar E.A., Bonfiglio M. (1987). The malignant potential of enchondromatosis. J. Bone Jt. Surg. Am..

[24] Aycan O.E., Sebastiani E., Bianchi G., Gambarotti M. (2019). Coexistence of secondary chondrosarcoma and lung carcinoma metastasis in the humerus of a patient with Ollier’s disease: A case report. Acta Orthop. Traumatol. Turc..

[25] Gonzalez M.R., Bryce-Alberti M., Portmann-Baracco A., Inchaustegui M.L., Castillo-Flores S., Pretell-Mazzini J. (2022). Appendicular dedifferentiated chondrosarcoma: A management and survival study from the SEER database. J. Bone Oncol..

[26] Desai K., Liu S., Baskovich B., Makary R. (2022). A Case Report and Brief Literature Review on Dedifferentiated Chondrosarcoma in Proximal Phalanx: A Rare Location. Cureus.

[27] Graham T.M. IHeartPathology Dedifferentiated Chondrosarcoma. https://www.iheartpathology.net/.

[28] Rozeman L.B., Hogendoorn P.C., Bovée J.V. (2002). Diagnosis and prognosis of chondrosarcoma of bone. Expert. Rev. Mol. Diagn..

[29] Malchenko S., Seftor E.A., Nikolsky Y., Hasegawa S.L., Kuo S., Stevens J.W., Poyarkov S., Nikolskaya T., Kucaba T., Wang M. (2012). Putative multifunctional signature of lung metastases in dedifferentiated chondrosarcoma. Sarcoma.

[30] Nguyen M.T., Jiang Y.Q., Li X.L., Dong J. (2019). Risk Factors for Incidence and Prognosis in Chondrosarcoma Patients with Pulmonary Metastasis at Initial Diagnosis. Med. Sci. Monit..

[31] Hung Y.P., Chebib I., Bredella M.A., Berner E.A., Taylor-Black Q., Choy E., Cote G.M., Chen Y.L., MacDonald S.M., Schwab J.H. (2023). Prognostic Significance of Percentage and Size of Dedifferentiation in Dedifferentiated Chondrosarcoma. Mod. Pathol..

[32] Badyal R.K., Kataria A.S., Kaur M. (2012). Primary chondrosarcoma of male breast: A rare case. Indian J. Surg..

[33] Albergo J.I., Gaston C.L., Jeys L.M., Khajuria A., Carter S.R., Tillman R.M., Abudu A.T., Grimer R.J. (2015). Management and prognostic significance of pathological fractures through chondrosarcoma of the femur. Int. Orthop..

[34] Bharath G., Burrah R., Shivakumar K., Manjunath S., Bhanumathi R. (2015). Dedifferentiated chondrosarcoma: An aggressive variant of chondrosarcoma. Asian Cardiovasc. Thorac. Ann..

[35] Douis H., Saifuddin A. (2012). The imaging of cartilaginous bone tumours. II. Chondrosarcoma. Skelet. Radiol..

[36] Littrell L.A., Wenger D.E., Wold L.E., Bertoni F., Unni K.K., White L.M., Kandel R., Sundaram M. (2004). Radiographic, CT, and MR Imaging Features of Dedifferentiated Chondrosarcomas: A Retrospective Review of 174 De Novo Cases. RadioGraphics.

[37] Kim J.-H., Lee S.K. (2023). Classification of Chondrosarcoma: From Characteristic to Challenging Imaging Findings. Cancers.

[38] MacSweeney F., Darby A., Saifuddin A. (2003). Dedifferentiated chondrosarcoma of the appendicular skeleton: MRI-pathological correlation. Skelet. Radiol..

[39] Tlemsani C., Larousserie F., De Percin S., Audard V., Hadjadj D., Chen J., Biau D., Anract P., Terris B., Goldwasser F. (2023). Biology and Management of High-Grade Chondrosarcoma: An Update on Targets and Treatment Options. Int. J. Mol. Sci..

[40] Gazendam A., Popovic S., Parasu N., Ghert M. (2023). Chondrosarcoma: A Clinical Review. J. Clin. Med..

[41] Saifuddin A., Mitchell R., Burnett S.J., Sandison A., Pringle J.A. (2000). Ultrasound-guided needle biopsy of primary bone tumours. J. Bone Jt. Surg. Br..

[42] Leddy L.R., Holmes R.E. (2014). Chondrosarcoma of bone. Cancer Treat. Res..

[43] Andreas F Mavrogenis P.J.P. Bone: Dedifferentiated Chondrosarcoma. Atlas of Genetics and Cytogenetics in Oncology and Haematology. https://atlasgeneticsoncology.org/solid-tumor/5063/bone-chondrosarcoma.

[44] Jelinek J.S., Murphey M.D., Welker J.A., Henshaw R.M., Kransdorf M.J., Shmookler B.M., Malawer M.M. (2002). Diagnosis of primary bone tumors with image-guided percutaneous biopsy: Experience with 110 tumors. Radiology.

[45] Altuntas A.O., Slavin J., Smith P.J., Schlict S.M., Powell G.J., Ngan S., Toner G., Choong P.F. (2005). Accuracy of computed tomography guided core needle biopsy of musculoskeletal tumours. ANZ J. Surg..

[46] Omura M.C., Motamedi K., UyBico S., Nelson S.D., Seeger L.L. (2011). Revisiting CT-guided percutaneous core needle biopsy of musculoskeletal lesions: Contributors to biopsy success. AJR Am. J. Roentgenol..

[47] Toki S., Sone M., Yoshida A., Nishisho T., Gokita T., Kobayashi E., Nakatani F., Chuman H., Sugawara S., Arai Y. (2022). Image-guided core needle biopsy for musculoskeletal lesions. J. Orthop. Sci..

[48] Dupuy D.E., Rosenberg A.E., Punyaratabandhu T., Tan M.H., Mankin H.J. (1998). Accuracy of CT-guided needle biopsy of musculoskeletal neoplasms. AJR Am. J. Roentgenol..

[49] Tanaka K., Ozaki T. (2019). New TNM classification (AJCC eighth edition) of bone and soft tissue sarcomas: JCOG Bone and Soft Tissue Tumor Study Group. Jpn. J. Clin. Oncol..

[50] Sakamoto A. (2014). The molecular pathogenesis of dedifferentiated chondrosarcoma. Indian. J. Orthop..

[51] Ropke M., Boltze C., Neumann H.W., Roessner A., Schneider-Stock R. (2003). Genetic and epigenetic alterations in tumor progression in a dedifferentiated chondrosarcoma. Pathol. Res. Pr..

[52] Dornauer K., Soder S., Inwards C.Y., Bovee J.V., Aigner T. (2010). Matrix biochemistry and cell biology of dedifferentiated chondrosarcomas. Pathol. Int..

[53] Meister P., Konrad E.A., Nathrath W., Eder M. (1980). Malignant fibrous histiocytoma: Histological patterns and cell types. Pathol. Res. Pr..

[54] Meijer D., de Jong D., Pansuriya T.C., van den Akker B.E., Picci P., Szuhai K., Bovee J.V. (2012). Genetic characterization of mesenchymal, clear cell, and dedifferentiated chondrosarcoma. Genes. Chromosom. Cancer.

[55] Yang L., Chen Q., Zhang S., Wang X., Li W., Wen J., Huang X., Zheng J., Huang G., Huang T. (2009). A novel mutated cell line with characteristics of dedifferentiated chondrosarcoma. Int. J. Mol. Med..

[56] Kozawa E., Nishida Y., Kawai A., Hayakawa K., Setsu N., Kawashima H., Iwata S., Tsuchiya H., Tsukushi S., Takenaka S. (2022). Clinical features and treatment outcomes of dedifferentiated and grade 3 chondrosarcoma: A multi-institutional study. Cancer Sci..

[57] Simard F.A., Richert I., Vandermoeten A., Decouvelaere A.V., Michot J.P., Caux C., Blay J.Y., Dutour A. (2016). Description of the immune microenvironment of chondrosarcoma and contribution to progression. Oncoimmunology.

[58] Singh A., Thorpe S.W., Darrow M., Carr-Ascher J.R. (2022). Case report: Treatment of metastatic dedifferentiated chondrosarcoma with pembrolizumab yields sustained complete response. Front. Oncol..

[59] Bingle L., Brown N.J., Lewis C.E. (2002). The role of tumour-associated macrophages in tumour progression: Implications for new anticancer therapies. J. Pathol..

[60] Pollard J.W. (2004). Tumour-educated macrophages promote tumour progression and metastasis. Nat. Rev. Cancer.

[61] Tawbi H.A., Burgess M., Bolejack V., Van Tine B.A., Schuetze S.M., Hu J., D’Angelo S., Attia S., Riedel R.F., Priebat D.A. (2017). Pembrolizumab in advanced soft-tissue sarcoma and bone sarcoma (SARC028): A multicentre, two-cohort, single-arm, open-label, phase 2 trial. Lancet Oncol..

[62] Kim J.R., Moon Y.J., Kwon K.S., Bae J.S., Wagle S., Kim K.M., Park H.S., Lee H., Moon W.S., Chung M.J. (2013). Tumor infiltrating PD1-positive lymphocytes and the expression of PD-L1 predict poor prognosis of soft tissue sarcomas. PLoS ONE.

[63] D’Angelo S.P., Shoushtari A.N., Agaram N.P., Kuk D., Qin L.X., Carvajal R.D., Dickson M.A., Gounder M., Keohan M.L., Schwartz G.K. (2015). Prevalence of tumor-infiltrating lymphocytes and PD-L1 expression in the soft tissue sarcoma microenvironment. Hum. Pathol..

[64] Kostine M., Cleven A.H., de Miranda N.F., Italiano A., Cleton-Jansen A.M., Bovee J.V. (2016). Analysis of PD-L1, T-cell infiltrate and HLA expression in chondrosarcoma indicates potential for response to immunotherapy specifically in the dedifferentiated subtype. Mod. Pathol..

[65] Karamchandani J.R., Nielsen T.O., van de Rijn M., West R.B. (2012). Sox10 and S100 in the diagnosis of soft-tissue neoplasms. Appl. Immunohistochem. Mol. Morphol..

[66] Bresnick A.R., Weber D.J., Zimmer D.B. (2015). S100 proteins in cancer. Nat. Rev. Cancer.

[67] Chebib I., Hornicek F.J., Bredella M.A., Deshpande V., Nielsen G.P. (2014). Histologic variants of chondrosarcoma. Diagn. Histopath..

[68] Thoenen E., Curl A., Iwakuma T. (2019). TP53 in bone and soft tissue sarcomas. Pharmacology.

[69] Terek R.M., Healey J.H., Garin-Chesa P., Mak S., Huvos A., Albino A.P. (1998). p53 mutations in chondrosarcoma. Diagn. Mol. Pathol..

[70] Endo M., de Graaff M.A., Ingram D.R., Lim S., Lev D.C., Briaire-de Bruijn I.H., Somaiah N., Bovee J.V., Lazar A.J., Nielsen T.O. (2015). NY-ESO-1 (CTAG1B) expression in mesenchymal tumors. Mod. Pathol..

[71] Lai J.P., Robbins P.F., Raffeld M., Aung P.P., Tsokos M., Rosenberg S.A., Miettinen M.M., Lee C.C. (2012). NY-ESO-1 expression in synovial sarcoma and other mesenchymal tumors: Significance for NY-ESO-1-based targeted therapy and differential diagnosis. Mod. Pathol..

[72] Han S., Liu Y., Cai S.J., Qian M., Ding J., Larion M., Gilbert M.R., Yang C. (2020). IDH mutation in glioma: Molecular mechanisms and potential therapeutic targets. Br. J. Cancer.

[73] Zhao S., Lin Y., Xu W., Jiang W., Zha Z., Wang P., Yu W., Li Z., Gong L., Peng Y. (2009). Glioma-derived mutations in IDH1 dominantly inhibit IDH1 catalytic activity and induce HIF-1alpha. Science.

[74] Abou-Alfa G.K., Macarulla T., Javle M.M., Kelley R.K., Lubner S.J., Adeva J., Cleary J.M., Catenacci D.V., Borad M.J., Bridgewater J. (2020). Ivosidenib in IDH1-mutant, chemotherapy-refractory cholangiocarcinoma (ClarIDHy): A multicentre, randomised, double-blind, placebo-controlled, phase 3 study. Lancet Oncol..

[75] Mardis E.R., Ding L., Dooling D.J., Larson D.E., McLellan M.D., Chen K., Koboldt D.C., Fulton R.S., Delehaunty K.D., McGrath S.D. (2009). Recurring mutations found by sequencing an acute myeloid leukemia genome. N. Engl. J. Med..

[76] Tallegas M., Miquelestorena-Standley E., Labit-Bouvier C., Badoual C., Francois A., Gomez-Brouchet A., Aubert S., Collin C., Tallet A., de Pinieux G. (2019). IDH mutation status in a series of 88 head and neck chondrosarcomas: Different profile between tumors of the skull base and tumors involving the facial skeleton and the laryngotracheal tract. Hum. Pathol..

[77] Tap W.D., Villalobos V.M., Cote G.M., Burris H., Janku F., Mir O., Beeram M., Wagner A.J., Jiang L., Wu B. (2020). Phase I Study of the Mutant IDH1 Inhibitor Ivosidenib: Safety and Clinical Activity in Patients with Advanced Chondrosarcoma. J. Clin. Oncol..

[78] Amary M.F., Bacsi K., Maggiani F., Damato S., Halai D., Berisha F., Pollock R., O’Donnell P., Grigoriadis A., Diss T. (2011). IDH1 and IDH2 mutations are frequent events in central chondrosarcoma and central and periosteal chondromas but not in other mesenchymal tumours. J. Pathol..

[79] Chen S., Fritchie K., Wei S., Ali N., Curless K., Shen T., Brini A.T., Latif F., Sumathi V., Siegal G.P. (2017). Diagnostic utility of IDH1/2 mutations to distinguish dedifferentiated chondrosarcoma from undifferentiated pleomorphic sarcoma of bone. Hum. Pathol..

[80] Yang T., Bai Y., Chen J., Sun K., Luo Y., Huang W., Zhang H. (2020). Clonality analysis and IDH1 and IDH2 mutation detection in both components of dedifferentiated chondrosarcoma, implicated its monoclonal origin. J. Bone Oncol..

[81] Dermawan J.K.T., Nafa K., Mohanty A., Xu Y., Rijo I., Casanova J., Villafania L., Benhamida J., Kelly C.M., Tap W.D. (2023). Distinct IDH1/2-associated Methylation Profile and Enrichment of TP53 and TERT Mutations Distinguish Dedifferentiated Chondrosarcoma from Conventional Chondrosarcoma. Cancer Res. Commun..

[82] Mak I.W., Singh S., Turcotte R., Ghert M. (2015). The epigenetic regulation of SOX9 by miR-145 in human chondrosarcoma. J. Cell. Biochem..

[83] Tang X., Lu X., Guo W., Ren T., Zhao H., Zhao F., Tang G. (2010). Different expression of Sox9 and Runx2 between chondrosarcoma and dedifferentiated chondrosarcoma cell line. Eur. J. Cancer Prev..

[84] Grote H.J., Schneider-Stock R., Neumann W., Roessner A. (2000). Mutation of p53 with loss of heterozygosity in the osteosarcomatous component of a dedifferentiated chondrosarcoma. Virchows Arch..

[85] Simms W.W., Ordonez N.G., Johnston D., Ayala A.G., Czerniak B. (1995). p53 expression in dedifferentiated chondrosarcoma. Cancer.

[86] Knosel T., Werner M., Jung A., Kirchner T., Durr H.R. (2014). Dedifferentiated chondrosarcoma mimicking a giant cell tumor. Is this low grade dedifferentiated chondrosarcoma?. Pathol. Res. Pr..

[87] Bovee J.V., Cleton-Jansen A.M., Rosenberg C., Taminiau A.H., Cornelisse C.J., Hogendoorn P.C. (1999). Molecular genetic characterization of both components of a dedifferentiated chondrosarcoma, with implications for its histogenesis. J. Pathol..

[88] Gilbert A. (2023). Chondrosarcoma Resistance to Radiation Therapy: Origins and Potential Therapeutic Solutions. Cancers.

[89] van Oosterwijk J.G., Meijer D., van Ruler M.A., van den Akker B.E., Oosting J., Krenacs T., Picci P., Flanagan A.M., Liegl-Atzwanger B., Leithner A. (2013). Screening for potential targets for therapy in mesenchymal, clear cell, and dedifferentiated chondrosarcoma reveals Bcl-2 family members and TGFbeta as potential targets. Am. J. Pathol..

[90] Franchi A., Baroni G., Sardi I., Giunti L., Capanna R., Campanacci D. (2012). Dedifferentiated peripheral chondrosarcoma: A clinicopathologic, immunohistochemical, and molecular analysis of four cases. Virchows Arch..

[91] Makise N., Sekimizu M., Konishi E., Motoi T., Kubo T., Ikoma H., Watanabe S.I., Okuma T., Hiraoka N., Fukayama M. (2019). H3K27me3 deficiency defines a subset of dedifferentiated chondrosarcomas with characteristic clinicopathological features. Mod. Pathol..

[92] Zajac A.E., Kopec S., Szostakowski B., Spalek M.J., Fiedorowicz M., Bylina E., Filipowicz P., Szumera-Cieckiewicz A., Tysarowski A., Czarnecka A.M. (2021). Chondrosarcoma-from Molecular Pathology to Novel Therapies. Cancers.

[93] Aigner T., Dertinger S., Belke J., Kirchner T. (1996). Chondrocytic cell differentiation in clear cell chondrosarcoma. Hum. Pathol..

[94] Oakley G.J., Fuhrer K., Seethala R.R. (2008). Brachyury, SOX-9, and podoplanin, new markers in the skull base chordoma vs chondrosarcoma differential: A tissue microarray-based comparative analysis. Mod. Pathol..

[95] Jeong W., Kim H.J. (2018). Biomarkers of chondrosarcoma. J. Clin. Pathol..

[96] Daugaard S., Christensen L.H., Hogdall E. (2009). Markers aiding the diagnosis of chondroid tumors: An immunohistochemical study including osteonectin, bcl-2, cox-2, actin, calponin, D2-40 (podoplanin), mdm-2, CD117 (c-kit), and YKL-40. APMIS.

[97] Syed M., Mushtaq S., Loya A., Hassan U. (2021). NKX3.1 a useful marker for mesenchymal chondrosarcoma: An immunohistochemical study. Ann. Diagn. Pathol..

[98] Kim M.J., Cho K.J., Ayala A.G., Ro J.Y. (2011). Chondrosarcoma: With updates on molecular genetics. Sarcoma.

[99] Folpe A.L., Graham R.P., Martinez A., Schembri-Wismayer D., Boland J., Fritchie K.J. (2018). Mesenchymal chondrosarcomas showing immunohistochemical evidence of rhabdomyoblastic differentiation: A potential diagnostic pitfall. Hum. Pathol..

[100] Fanburg-Smith J.C., Auerbach A., Marwaha J.S., Wang Z., Santi M., Judkins A.R., Rushing E.J. (2010). Immunoprofile of mesenchymal chondrosarcoma: Aberrant desmin and EMA expression, retention of INI1, and negative estrogen receptor in 22 female-predominant central nervous system and musculoskeletal cases. Ann. Diagn. Pathol..

[101] Estrada-Villaseñor E., Rico-Martínez G., Linares-Gonzalez L.M. (2010). Diagnosis of a dedifferentiated chondrosarcoma of the pelvis by fine needle aspiration. A case report. Acta Cytol..

[102] Sobti A., Agrawal P., Agarwala S., Agarwal M. (2016). Giant Cell Tumor of Bone—An Overview. Arch. Bone Jt. Surg..

[103] Wick M.R., Siegal G.P., Mills S.E., Thompson R.C., Sawhney D., Fechner R.E. (1987). Dedifferentiated chondrosarcoma of bone. An immunohistochemical and lectin-histochemical study. Virchows Arch. A Pathol. Anat. Histopathol..

[104] Bahrami A., Truong L.D., Ro J.Y. (2008). Undifferentiated tumor: True identity by immunohistochemistry. Arch. Pathol. Lab. Med..

[105] Yoshida A., Ushiku T., Motoi T., Beppu Y., Fukayama M., Tsuda H., Shibata T. (2012). MDM2 and CDK4 immunohistochemical coexpression in high-grade osteosarcoma: Correlation with a dedifferentiated subtype. Am. J. Surg. Pathol..

[106] Junior A.T., de Abreu Alves F., Pinto C.A., Carvalho A.L., Kowalski L.P., Lopes M.A. (2003). Clinicopathological and immunohistochemical analysis of twenty-five head and neck osteosarcomas. Oral. Oncol..

[107] Al-Khan A.A., Gunn H.J., Day M.J., Tayebi M., Ryan S.D., Kuntz C.A., Saad E.S., Richardson S.J., Danks J.A. (2017). Immunohistochemical Validation of Spontaneously Arising Canine Osteosarcoma as a Model for Human Osteosarcoma. J. Comp. Pathol..

[108] Mardanpour K., Rahbar M., Mardanpour S. (2016). Coexistence of HER2, Ki67, and p53 in Osteosarcoma: A Strong Prognostic Factor. N. Am. J. Med. Sci..

[109] Barger A., Graca R., Bailey K., Messick J., de Lorimier L.P., Fan T., Hoffmann W. (2005). Use of alkaline phosphatase staining to differentiate canine osteosarcoma from other vimentin-positive tumors. Vet. Pathol..

[110] Thway K. (2009). Pathology of soft tissue sarcomas. Clin. Oncol. R. Coll. Radiol..

[111] Augsburger D., Nelson P.J., Kalinski T., Udelnow A., Knosel T., Hofstetter M., Qin J.W., Wang Y., Gupta A.S., Bonifatius S. (2017). Current diagnostics and treatment of fibrosarcoma -perspectives for future therapeutic targets and strategies. Oncotarget.

[112] Folpe A.L. (2014). Fibrosarcoma: A review and update. Histopathology.

[113] Meis-Kindblom J.M., Kindblom L.G., Enzinger F.M. (1995). Sclerosing epithelioid fibrosarcoma. A variant of fibrosarcoma simulating carcinoma. Am. J. Surg. Pathol..

[114] Munday J.S., Stedman N.L., Richey L.J. (2003). Histology and immunohistochemistry of seven ferret vaccination-site fibrosarcomas. Vet. Pathol..

[115] Oda Y., Miyajima K., Kawaguchi K., Tamiya S., Oshiro Y., Hachitanda Y., Oya M., Iwamoto Y., Tsuneyoshi M. (2001). Pleomorphic leiomyosarcoma: Clinicopathologic and immunohistochemical study with special emphasis on its distinction from ordinary leiomyosarcoma and malignant fibrous histiocytoma. Am. J. Surg. Pathol..

[116] Coindre J.M., Mariani O., Chibon F., Mairal A., De Saint Aubain Somerhausen N., Favre-Guillevin E., Bui N.B., Stoeckle E., Hostein I., Aurias A. (2003). Most malignant fibrous histiocytomas developed in the retroperitoneum are dedifferentiated liposarcomas: A review of 25 cases initially diagnosed as malignant fibrous histiocytoma. Mod. Pathol..

[117] Lawson C.W., Fisher C., Gatter K.C. (1987). An immunohistochemical study of differentiation in malignant fibrous histiocytoma. Histopathology.

[118] Al-Agha O.M., Igbokwe A.A. (2008). Malignant fibrous histiocytoma: Between the past and the present. Arch. Pathol. Lab. Med..

[119] Coindre J.M. (2003). Immunohistochemistry in the diagnosis of soft tissue tumours. Histopathology.

[120] Machado I., Mayordomo-Aranda E., Giner F., Llombart-Bosch A. (2015). The Role of Immunohistochemistry in Rhabdomyosarcoma Diagnosis Using Tissue Microarray Technology and a Xenograft Model. Fetal Pediatr. Pathol..

[121] Wachtel M., Runge T., Leuschner I., Stegmaier S., Koscielniak E., Treuner J., Odermatt B., Behnke S., Niggli F.K., Schafer B.W. (2006). Subtype and prognostic classification of rhabdomyosarcoma by immunohistochemistry. J. Clin. Oncol..

[122] Mentzel T., Kuhnen C. (2006). Spindle cell rhabdomyosarcoma in adults: Clinicopathological and immunohistochemical analysis of seven new cases. Virchows Arch..

[123] Carvalho J.C., Thomas D.G., Lucas D.R. (2009). Cluster analysis of immunohistochemical markers in leiomyosarcoma delineates specific anatomic and gender subgroups. Cancer.

[124] Mills A.M., Ly A., Balzer B.L., Hendrickson M.R., Kempson R.L., McKenney J.K., Longacre T.A. (2013). Cell cycle regulatory markers in uterine atypical leiomyoma and leiomyosarcoma: Immunohistochemical study of 68 cases with clinical follow-up. Am. J. Surg. Pathol..

[125] Lugowska I., Teterycz P., Mikula M., Kulecka M., Kluska A., Balabas A., Piatkowska M., Wagrodzki M., Pienkowski A., Rutkowski P. (2018). IDH1/2 Mutations Predict Shorter Survival in Chondrosarcoma. J. Cancer.

[126] Nakagawa M., Sekimizu M., Endo M., Kobayashi E., Iwata S., Fukushima S., Yoshida A., Kitabayashi I., Ichikawa H., Kawai A. (2022). Prognostic impact of IDH mutations in chondrosarcoma. J. Orthop. Sci..

[127] Vuong H.G., Ngo T.N.M., Dunn I.F. (2021). Prognostic importance of IDH mutations in chondrosarcoma: An individual patient data meta-analysis. Cancer Med..

[128] Li Y., Yang S., Liu Y., Yang S. (2022). Mice with Trp53 and Rb1 deficiency in chondrocytes spontaneously develop chondrosarcoma via overactivation of YAP signaling. Cell Death Dis..

[129] Venneker S., Kruisselbrink A.B., Baranski Z., Palubeckaite I., Briaire-de Bruijn I.H., Oosting J., French P.J., Danen E.H.J., Bovee J. (2020). Beyond the Influence of IDH Mutations: Exploring Epigenetic Vulnerabilities in Chondrosarcoma. Cancers.

[130] Nakagawa M., Yamaguchi M., Endo M., Machida Y., Hattori A., Tanzawa F., Tsutsumi S., Kitabayashi I., Kawai A., Nakatani F. (2022). Clinical usefulness of 2-hydroxyglutarate as a biomarker in IDH-mutant chondrosarcoma. J. Bone Oncol..

[131] Bovée J.V.M.G., Bloem J.L., Flanagan A.M., Nielsen G.P., Yoshida A. (2020). WHO Classification of Tumours: Soft Tissue and Bone Tumours.

[132] Nicolle R., Ayadi M., Gomez-Brouchet A., Armenoult L., Banneau G., Elarouci N., Tallegas M., Decouvelaere A.V., Aubert S., Redini F. (2019). Integrated molecular characterization of chondrosarcoma reveals critical determinants of disease progression. Nat. Commun..

[133] Miwa S., Yamamoto N., Hayashi K., Takeuchi A., Igarashi K., Tsuchiya H. (2022). Therapeutic Targets and Emerging Treatments in Advanced Chondrosarcoma. Int. J. Mol. Sci..

[134] Zhu G., Pan C., Bei J.X., Li B., Liang C., Xu Y., Fu X. (2020). Mutant p53 in Cancer Progression and Targeted Therapies. Front. Oncol..

[135] Sandberg A.A. (2004). Genetics of chondrosarcoma and related tumors. Curr. Opin. Oncol..

[136] Lucas C.G., Grenert J.P., Horvai A. (2021). Targeted Next-Generation Sequencing Identifies Molecular and Genetic Events in Dedifferentiated Chondrosarcoma. Arch. Pathol. Lab. Med..

[137] Nazeri E., Gouran Savadkoohi M., Majidzadeh A.K., Esmaeili R. (2018). Chondrosarcoma: An overview of clinical behavior, molecular mechanisms mediated drug resistance and potential therapeutic targets. Crit. Rev. Oncol. Hematol..

[138] Oshiro Y., Chaturvedi V., Hayden D., Nazeer T., Johnson M., Johnston D.A., Ordez N.G., Ayala A.G., Czerniak B. (1998). Altered p53 is associated with aggressive behavior of chondrosarcoma. Cancer.

[139] Suzuki H., Zhou X., Yin J., Lei J., Jiang H.Y., Suzuki Y., Chan T., Hannon G.J., Mergner W.J., Abraham J.M. (1995). Intragenic mutations of CDKN2B and CDKN2A in primary human esophageal cancers. Hum. Mol. Genet..

[140] Tarpey P.S., Behjati S., Cooke S.L., Van Loo P., Wedge D.C., Pillay N., Marshall J., O’Meara S., Davies H., Nik-Zainal S. (2013). Frequent mutation of the major cartilage collagen gene COL2A1 in chondrosarcoma. Nat. Genet..

[141] Amary M.F., Ye H., Forbes G., Damato S., Maggiani F., Pollock R., Tirabosco R., Flanagan A.M. (2015). Isocitrate dehydrogenase 1 mutations (IDH1) and p16/CDKN2A copy number change in conventional chondrosarcomas. Virchows Arch..

[142] Chow W.A. (2018). Chondrosarcoma: Biology, genetics, and epigenetics. F1000Research.

[143] Gao L., Hong X., Guo X., Cao D., Gao X., DeLaney T.F., Gong X., Chen R., Ni J., Yao Y. (2016). Targeted next-generation sequencing of dedifferentiated chondrosarcoma in the skull base reveals combined TP53 and PTEN mutations with increased proliferation index, an implication for pathogenesis. Oncotarget.

[144] O’Malley D.P., Opheim K.E., Barry T.S., Chapman D.B., Emond M.J., Conrad E.U., Norwood T.H. (2001). Chromosomal changes in a dedifferentiated chondrosarcoma: A case report and review of the literature. Cancer Genet. Cytogenet..

[145] Kattepur A.K., Jones R.L., Gulia A. (2021). Dedifferentiated chondrosarcoma: Current standards of care. Future Oncol..

[146] Stevenson J.D., Laitinen M.K., Parry M.C., Sumathi V., Grimer R.J., Jeys L.M. (2018). The role of surgical margins in chondrosarcoma. Eur. J. Surg. Oncol..

[147] Deloin X., Dumaine V., Biau D., Karoubi M., Babinet A., Tomeno B., Anract P. (2009). Pelvic chondrosarcomas: Surgical treatment options. Orthop. Traumatol. Surg. Res..

[148] Bruns J., Fiedler W., Werner M., Delling G. (2005). Dedifferentiated chondrosarcoma—A fatal disease. J. Cancer Res. Clin. Oncol..

[149] Sambri A., Tuzzato G., Donati D.M., De Paolis M., Bianchi G. (2021). Pathological fracture does not affect prognosis in dedifferentiated chondrosarcoma of the limbs. J. Orthop. Sci..

[150] Walter S.G., Knöll P., Eysel P., Quaas A., Gaisendrees C., Nißler R., Hieggelke L. (2023). Molecular In-Depth Characterization of Chondrosarcoma for Current and Future Targeted Therapies. Cancers.

[151] Ollivier L., Vanel D., Leclere J. (2003). Imaging of chondrosarcomas. Cancer Imaging.

[152] Gomez C.D., Anderson M.S., Epperly S.C., Zuckerman L.M. (2020). Successful treatment of a dedifferentiated chondrosarcoma of the proximal humerus with a hemicortical articular surface sparing allograft: A case report. Int. J. Surg. Case Rep..

[153] Davies B.W., Prescott C.R., Said S.A., Campana J., Attie-Castro F.A., Velasco E.C.A.A., Durairaj V.D. (2014). Radiation-induced dedifferentiated chondrosarcoma with orbital invasion. Ophthalmic Plast. Reconstr. Surg..

[154] Coskun H.S., Erdogan F., Buyukceran I., Dabak N. (2022). Evaluation of prognostic factors affecting survival in chondrosarcoma treatment and comparison with literature. Jt. Dis. Relat. Surg..

[155] Kremenevski N., Schlaffer S.M., Coras R., Kinfe T.M., Graillon T., Buchfelder M. (2020). Skull Base Chordomas and Chondrosarcomas. Neuroendocrinology.

[156] Harwood A.R., Krajbich J.I., Fornasier V.L. (1980). Radiotherapy of chondrosarcoma of bone. Cancer.

[157] Krochak R., Harwood A.R., Cummings B.J., Quirt I.C. (1983). Results of radical radiation for chondrosarcoma of bone. Radiother. Oncol..

[158] Lex J.R., Evans S., Stevenson J.D., Parry M., Jeys L.M., Grimer R.J. (2018). Dedifferentiated chondrosarcoma of the pelvis: Clinical outcomes and current treatment. Clin. Sarcoma Res..

[159] Dickey I.D., Rose P.S., Fuchs B., Wold L.E., Okuno S.H., Sim F.H., Scully S.P. (2004). Dedifferentiated chondrosarcoma: The role of chemotherapy with updated outcomes. J. Bone Jt. Surg. Am..

[160] MacDonald I.J., Lin C.Y., Kuo S.J., Su C.M., Tang C.H. (2019). An update on current and future treatment options for chondrosarcoma. Expert. Rev. Anticancer..

[161] van Maldegem A., Conley A.P., Rutkowski P., Patel S.R., Lugowska I., Desar I.M.E., Bovée J., Gelderblom H. (2019). Outcome of First-Line Systemic Treatment for Unresectable Conventional, Dedifferentiated, Mesenchymal, and Clear Cell Chondrosarcoma. Oncologist.

[162] Bui N., Dietz H., Farag S., Hirbe A.C., Wagner M.J., Van Tine B.A., Ganjoo K., Jones R.L., Keedy V.L., Davis E.J. (2023). A Retrospective Multi-Institutional Cohort Analysis of Clinical Characteristics and Outcomes in Dedifferentiated Chondrosarcoma. Cancers.

[163] Frassica F.J., Unni K.K., Beabout J.W., Sim F.H. (1986). Dedifferentiated chondrosarcoma. A report of the clinicopathological features and treatment of seventy-eight cases. J. Bone Jt. Surg. Am..

[164] Sheth D.S., Yasko A.W., Johnson M.E., Ayala A.G., Murray J.A., Romsdahl M.M. (1996). Chondrosarcoma of the pelvis. Prognostic factors for 67 patients treated with definitive surgery. Cancer.

[165] Kawaguchi S., Sun T., Lin P.P., Deavers M., Harun N., Lewis V.O. (2014). Does ifosfamide therapy improve survival of patients with dedifferentiated chondrosarcoma?. Clin. Orthop. Relat. Res..

[166] Hompland I., Ferrari S., Bielack S., Palmerini E., Hall K.S., Picci P., Hecker-Nolting S., Donati D.M., Blattmann C., Bjerkehagen B. (2021). Outcome in dedifferentiated chondrosarcoma for patients treated with multimodal therapy: Results from the EUROpean Bone Over 40 Sarcoma Study. Eur. J. Cancer.

[167] Italiano A., Mir O., Cioffi A., Palmerini E., Piperno-Neumann S., Perrin C., Chaigneau L., Penel N., Duffaud F., Kurtz J.E. (2013). Advanced chondrosarcomas: Role of chemotherapy and survival. Ann. Oncol..

[168] Cranmer L.D., Chau B., Mantilla J.G., Loggers E.T., Pollack S.M., Kim T.S., Kim E.Y., Kane G.M., Thompson M.J., Harwood J.L. (2022). Is Chemotherapy Associated with Improved Overall Survival in Patients with Dedifferentiated Chondrosarcoma? A SEER Database Analysis. Clin. Orthop. Relat. Res..

[169] Mitchell A.D., Ayoub K., Mangham D.C., Grimer R.J., Carter S.R., Tillman R.M. (2000). Experience in the treatment of dedifferentiated chondrosarcoma. J. Bone Jt. Surg. Br..

[170] Streitbuerger A., Ahrens H., Gosheger G., Henrichs M., Balke M., Dieckmann R., Hardes J. (2012). The treatment of locally recurrent chondrosarcoma: Is extensive further surgery justified?. J. Bone Jt. Surg. Br..

[171] Iseulys R., Anne G.B., Corinne B., Gonzague D.B.P., Marie K., Jean-Yves B., Aurelie D. (2020). The immune landscape of chondrosarcoma reveals an immunosuppressive environment in the dedifferentiated subtypes and exposes CSFR1+ macrophages as a promising therapeutic target. J. Bone Oncol..

[172] Koirala P., Roth M.E., Gill J., Piperdi S., Chinai J.M., Geller D.S., Hoang B.H., Park A., Fremed M.A., Zang X. (2016). Immune infiltration and PD-L1 expression in the tumor microenvironment are prognostic in osteosarcoma. Sci. Rep..

[173] Wagner M.J., Ricciotti R.W., Mantilla J., Loggers E.T., Pollack S.M., Cranmer L.D. (2018). Response to PD1 inhibition in conventional chondrosarcoma. J. Immunother. Cancer.

[174] Paoluzzi L., Cacavio A., Ghesani M., Karambelkar A., Rapkiewicz A., Weber J., Rosen G. (2016). Response to anti-PD1 therapy with nivolumab in metastatic sarcomas. Clin. Sarcoma Res..

[175] Palmerini E., Lopez-Pousa A., Grignani G., Redondo A., Hindi N., Stacchiotti S., Sebio A., Lopez-Martin J.A., Morales C.M.V., Martinez-Trufero J. (2020). IMMUNOSARC: A collaborative Spanish (GEIS) and Italian (ISG) sarcoma groups phase I/II trial of sunitinib and nivolumab in advanced soft tissue and bone sarcoma: Results from the phase II part, bone sarcoma cohort. J. Clin. Oncol..

[176] Zhang Y.X., van Oosterwijk J.G., Sicinska E., Moss S., Remillard S.P., van Wezel T., Buhnemann C., Hassan A.B., Demetri G.D., Bovee J.V. (2013). Functional profiling of receptor tyrosine kinases and downstream signaling in human chondrosarcomas identifies pathways for rational targeted therapy. Clin. Cancer Res..

[177] Polychronidou G., Karavasilis V., Pollack S.M., Huang P.H., Lee A., Jones R.L. (2017). Novel therapeutic approaches in chondrosarcoma. Future Oncol..

[178] Schrage Y.M., Briaire-de Bruijn I.H., de Miranda N.F., van Oosterwijk J., Taminiau A.H., van Wezel T., Hogendoorn P.C., Bovee J.V. (2009). Kinome profiling of chondrosarcoma reveals SRC-pathway activity and dasatinib as option for treatment. Cancer Res..

[179] Bernstein-Molho R., Kollender Y., Issakov J., Bickels J., Dadia S., Flusser G., Meller I., Sagi-Eisenberg R., Merimsky O. (2012). Clinical activity of mTOR inhibition in combination with cyclophosphamide in the treatment of recurrent unresectable chondrosarcomas. Cancer Chemother. Pharm..

[180] Micaily I., Roche M., Ibrahim M.Y., Martinez-Outschoorn U., Mallick A.B. (2021). Metabolic Pathways and Targets in Chondrosarcoma. Front. Oncol..

[181] Grignani G., Palmerini E., Stacchiotti S., Boglione A., Ferraresi V., Frustaci S., Comandone A., Casali P.G., Ferrari S., Aglietta M. (2011). A phase 2 trial of imatinib mesylate in patients with recurrent nonresectable chondrosarcomas expressing platelet-derived growth factor receptor-alpha or -beta: An Italian Sarcoma Group study. Cancer.

[182] Schuetze S.M., Bolejack V., Choy E., Ganjoo K.N., Staddon A.P., Chow W.A., Tawbi H.A., Samuels B.L., Patel S.R., von Mehren M. (2017). Phase 2 study of dasatinib in patients with alveolar soft part sarcoma, chondrosarcoma, chordoma, epithelioid sarcoma, or solitary fibrous tumor. Cancer.

[183] Albarran V., Villamayor M.L., Chamorro J., Rosero D.I., Pozas J., San Roman M., Calvo J.C., Perez de Aguado P., Moreno J., Guerrero P. (2022). Receptor Tyrosine Kinase Inhibitors for the Treatment of Recurrent and Unresectable Bone Sarcomas. Int. J. Mol. Sci..

[184] Tian W., Zhang W., Wang Y., Jin R., Wang Y., Guo H., Tang Y., Yao X. (2022). Recent advances of IDH1 mutant inhibitor in cancer therapy. Front. Pharmacol..

[185] Italiano A., Le Cesne A., Bellera C., Piperno-Neumann S., Duffaud F., Penel N., Cassier P., Domont J., Takebe N., Kind M. (2013). GDC-0449 in patients with advanced chondrosarcomas: A French Sarcoma Group/US and French National Cancer Institute Single-Arm Phase II Collaborative Study. Ann. Oncol..

[186] Lin Z.S., Chung C.C., Liu Y.C., Chang C.H., Liu H.C., Liang Y.Y., Huang T.L., Chen T.M., Lee C.H., Tang C.H. (2023). EZH2/hSULF1 axis mediates receptor tyrosine kinase signaling to shape cartilage tumor progression. Elife.

[187] M.D. Anderson Cancer Center LN-145 or LN-145-S1 in Treating Patients with Relapsed or Refractory Ovarian Cancer, Triple Negative Breast Cancer (TNBC), Anaplastic Thyroid Cancer, Osteosarcoma, or Other Bone and Soft Tissue Sarcomas. https://classic.clinicaltrials.gov/ct2/show/NCT03449108.

[188] Gettinger S., Kluger H., Schoenfeld A., Warner A.B., He K., Sukari A., Thomas S.S., Spéville B.D.d., Lee S., Haefliger S. (2021). 187TiP Phase II, multicenter study of autologous tumor infiltrating lymphocytes (TIL, LN 144/LN-145/LN-145-S1) in patients with solid tumours. J. Thorac. Oncol..

[189] Biermann J.S., Chow W., Reed D.R., Lucas D., Adkins D.R., Agulnik M., Benjamin R.S., Brigman B., Budd T., Curry W.T. (2017). NCCN Guidelines Insights: Bone Cancer, Version 2.2017. J. Natl. Compr. Canc. Netw..

[190] Duffaud F., Mir O., Boudou-Rouquette P., Piperno-Neumann S., Penel N., Bompas E., Delcambre C., Kalbacher E., Italiano A., Collard O. (2019). Efficacy and safety of regorafenib in adult patients with metastatic osteosarcoma: A non-comparative, randomised, double-blind, placebo-controlled, phase 2 study. Lancet Oncol..

